# 5-HT_6_ receptor recruitment of mTOR as a mechanism for perturbed cognition in schizophrenia

**DOI:** 10.1002/emmm.201201410

**Published:** 2012-10-02

**Authors:** Julie Meffre, Séverine Chaumont-Dubel, Clotilde Mannoury la Cour, Florence Loiseau, David J G Watson, Anne Dekeyne, Martial Séveno, Jean-Michel Rivet, Florence Gaven, Paul Déléris, Denis Hervé, Kevin C F Fone, Joël Bockaert, Mark J Millan, Philippe Marin

**Affiliations:** 1 CNRS, UMR-5203, Institut de Génomique FonctionnelleMontpellier, France; 2 INSERM, U661Montpellier, France; 3 Universités Montpellier 1 & 2Montpellier, France; 4 Institut de Recherches ServierCroissy-sur-Seine, France; 5 School of Biomedical Sciences, Medical School, Queen's Medical Centre, The University of NottinghamNottingham, UK; 6 Institut du Fer à Moulin, INSERM, UMRS-839, UPMCParis, France

**Keywords:** 5-HT_6_ receptor, cognition, mTORC1, proteomics, schizophrenia

## Abstract

Cognitive deficits in schizophrenia severely compromise quality of life and are poorly controlled by current antipsychotics. While 5-HT_6_ receptor blockade holds special promise, molecular substrates underlying their control of cognition remain unclear. Using a proteomic strategy, we show that 5-HT_6_ receptors physically interact with several proteins of the mammalian target of rapamycin (mTOR) pathway, including mTOR. Further, 5-HT_6_ receptor activation increased mTOR signalling in rodent prefrontal cortex (PFC). Linking this signalling event to cognitive impairment, the mTOR inhibitor rapamycin prevented deficits in social cognition and novel object discrimination induced by 5-HT_6_ agonists. In two developmental models of schizophrenia, specifically neonatal phencyclidine treatment and post-weaning isolation rearing, the activity of mTOR was enhanced in the PFC, and rapamycin, like 5-HT_6_ antagonists, reversed these cognitive deficits. These observations suggest that recruitment of mTOR by prefrontal 5-HT_6_ receptors contributes to the perturbed cognition in schizophrenia, offering new vistas for its therapeutic control.

## INTRODUCTION

There is increasing recognition that cognitive deficits in schizophrenia severely compromise the real world function of patients. Despite their efficacy against positive symptoms, currently available antipsychotics are poorly effective against cognitive dysfunction. Accordingly, intensive efforts are being made to improve our understanding of pathological mechanisms underlying cognitive impairment in schizophrenia and to identify novel strategies for its alleviation (Gray & Roth, 2007; Insel, 2010; Millan et al, 2012).

Amongst mechanisms currently under study, 5-HT_6_ receptors are of special interest, in as much as their blockade consistently enhances mnemonic performance in a broad range of procedures in rodents (Hirst et al, 2006; Loiseau et al, 2008; Marcos et al, 2008; Rogers & Hagan, 2001; Woolley et al, 2001), while 5-HT_6_ receptor antagonists also promote the corticolimbic release of acetylcholine, glutamate and monoamines to favour cognitive processes (Codony et al, 2011). Further, there is preliminary evidence for pro-cognitive properties of 5-HT_6_ receptor antagonists in humans (Codony et al, 2011; Johnson et al, 2008; Mitchell & Neumaier, 2005). Nonetheless, the nature of signalling mechanisms mediating the influence of 5-HT_6_ receptors on cognition remains unclear, complicating analysis of their significance to the induction and control of cognitive deficits in schizophrenia and other disorders. 5-HT_6_ receptors stimulate Gs and adenylyl cyclase (Millan et al, 2008). They also activate extracellular-regulated kinase (ERK)1,2 via the Src-family tyrosine kinase Fyn (Yun et al, 2007). However, these pathways exert a positive influence on cognition (Millan et al, 2012). Hence, it is unlikely that their inactivation would transduce the pro-cognitive effects of 5-HT_6_ receptor antagonists, raising the possibility that alternative coupling mechanisms are involved.

In light of recent evidence that G protein-coupled receptors (GPCRs) can interact with extensive protein networks, including proteins dedicated to signal transduction (Bockaert et al, 2004), we used a proteomic strategy to identify proteins potentially associated with 5-HT_6_ receptors. Based on identification of several proteins of the mammalian target of rapamycin (mTOR) pathway (including mTOR itself, Raptor, Neurofibromin 1 and Vps34, a class III phosphatidyl inositol 3-kinase), we then undertook an extensive suite of *in vivo* studies to determine whether 5-HT_6_ receptor engagement of mTOR contributes to their deleterious influence upon cognition, specifically in developmental models of schizophrenia.

## RESULTS

### 5-HT_6_ receptors physically interact with the mTOR complex 1

**Figure 1:**
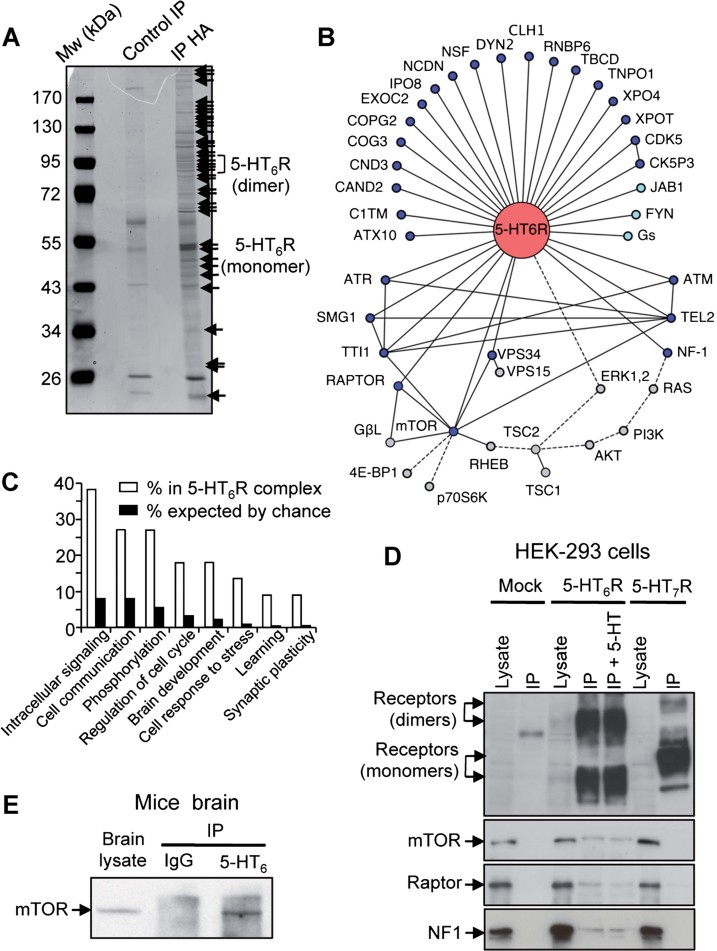
Proteomic characterization of the 5-HT_6_ receptor-associated complex SDS–PAGE analysis of proteins co-immunoprecipitated with HA-5-HT_6_ receptors expressed in HEK-293 cells. Control immunoprecipitations were performed in presence of 100 µM HA peptide. A colloidal Coomassie blue-stained gel obtained in a typical experiment is shown. Arrows indicate bands not detected in control immunoprecipitation.Graphic representation of the interactions between the 5-HT_6_ receptor and its protein partners. The red node corresponds to our bait, dark blue nodes to proteins identified as receptor partners in at least three out of four experiments, light blue nodes to already described 5-HT_6_ receptor partners and grey nodes to proteins of the mTOR pathway not identified in our screen. For edges attributes, full lines represent established interactions and dotted lines signalling pathways.GO annotations and functional enrichment were analysed using BINGO. Only categories that are more represented than expected by chance are shown.Analysis of mTOR, Raptor and Neurofibromin 1 (NF1) co-immunoprecipitation with HA-5HT_6_ receptors and HA-5-HT_7_ receptor expressed in HEK-293 cells by Western blotting. Control immunoprecipitation (Mock) was performed by using cells transfected with an empty vector. When indicated, cells were challenged for 2 min with 1 µM 5-HT.Co-immunoprecipitation of mTOR with native 5-HT_6_ receptors expressed in mice brain. Representative data of three independent experiments are illustrated in (**D**) and (**E)**.

Due to the low density of 5-HT_6_ receptors in mammalian brain and the lack of an antibody permitting immunoprecipitation yields compatible with mass spectrometry analysis, we purified receptor-interacting proteins by co-immunoprecipitation with a hemagglutinin (HA)-tagged 5-HT_6_ receptor expressed in human embryonic kidney (HEK)-293 cells. Functionality of HA-5-HT_6_ receptors was assessed by the ability of 5-HT and two synthetic 5-HT_6_ agonists, WAY181187 and WAY208466 (Schechter et al, 2008), to increase cAMP production (Supporting Information Fig S1). Analysis of affinity-purified proteins by SDS–PAGE revealed the presence of proteins that co-immunoprecipitated with the receptor and that were not detected in control immunoprecipitations performed in the presence of HA peptide (Fig 1A). Correspondingly, systematic analysis by high-resolution nanoflow liquid tandem mass spectrometry of gel lanes identified 28 proteins, which specifically co-immunoprecipitated with the 5-HT_6_ receptor (Fig 1B and Supporting Information Tables S1 and S2). These proteins were considered as potential partners of the receptor, though one cannot rule out the possibility that some of them do not interact with the 5-HT_6_ receptor but that their presence reflects some affinity for the anti-HA antibody. Compared with what would be expected by chance, the 5-HT_6_ receptor ‘interactome’ showed a remarkable enrichment in proteins implicated in intracellular signalling pathways, brain development, learning and synaptic plasticity (Fig 1C). These include several proteins of the mTOR pathway such as mTOR itself and Raptor, which together with GβL, constitute the rapamycin-sensitive mTOR complex 1 (mTORC1; Laplante & Sabatini, 2012; Swiech et al, 2008; Wang & Proud, 2011; Zhou & Huang, 2010). mTOR also forms the mTOR complex 2 (mTORC2), which includes specific members (Rictor, mSin1 and Protor1/2) in addition to mTOR and GβL but is insensitive to acute rapamycin treatment (Laplante & Sabatini, 2012; Swiech et al, 2008; Wang & Proud, 2011; Zhou & Huang, 2010). None of the proteins specific to mTORC2 were detected in the 5-HT_6_ receptor interactome, suggesting a specific recruitment of mTORC1 by this receptor. The 5-HT_6_ receptor also recruited Tti1 and Tel2, two proteins critical for assembly and activity of mTORC1 and 2 (Kaizuka et al, 2010). In addition, two proteins of the pathways leading to mTOR activation were identified: the Ras GTPase activating protein (GAP) Neurofibromin 1 and the class III phosphatidyl inositol 3-kinase Vps34 (Swiech et al, 2008; Zhou & Huang, 2010; Fig 1B). Immunoprecipitation followed by Western blot analysis confirmed the constitutive interaction of mTOR, Raptor and Neurofibromin 1 with the 5-HT_6_ receptor in HEK-293 cells and indicated that their recruitment was not further increased upon receptor activation by 5-HT (Fig 1D). Importantly, mTOR specifically co-immunoprecipitated with native 5-HT_6_ receptor expressed in mice brain (Fig 1E), indicating that they form a complex *in vivo*. Further supporting a specific association of mTORC1 with the 5-HT_6_ receptor, neither mTOR nor Raptor co-immunoprecipitated with the 5-HT_7_ receptor (another Gs-coupled 5-HT receptor) expressed in HEK-293 cells (Fig 1D). Likewise, mTOR did not co-immunoprecipitate with a truncated 5-HT_6_ receptor deleted of the 49 carboxy-terminal residues (5-HT_6_Δ49Ct, Supporting Information Fig S2), indicating a role of the 5-HT_6_ receptor C-terminal domain in its physical association with mTORC1. Nonetheless, other parts of the receptor sequence were necessary to mTORC1 recruitment as mTOR did not bind to the receptor C-terminus in GST pull-downs using the entire receptor C-terminal sequence as bait (Supporting Information Fig S2C).

### 5-HT_6_ receptors activate the mTOR pathway in HEK-293 cells via a mechanism requiring both the canonical phosphatidyl inositol 3-kinase/Akt/Rheb pathway and 5-HT_6_ receptor/mTOR physical interaction

**Figure 2:**
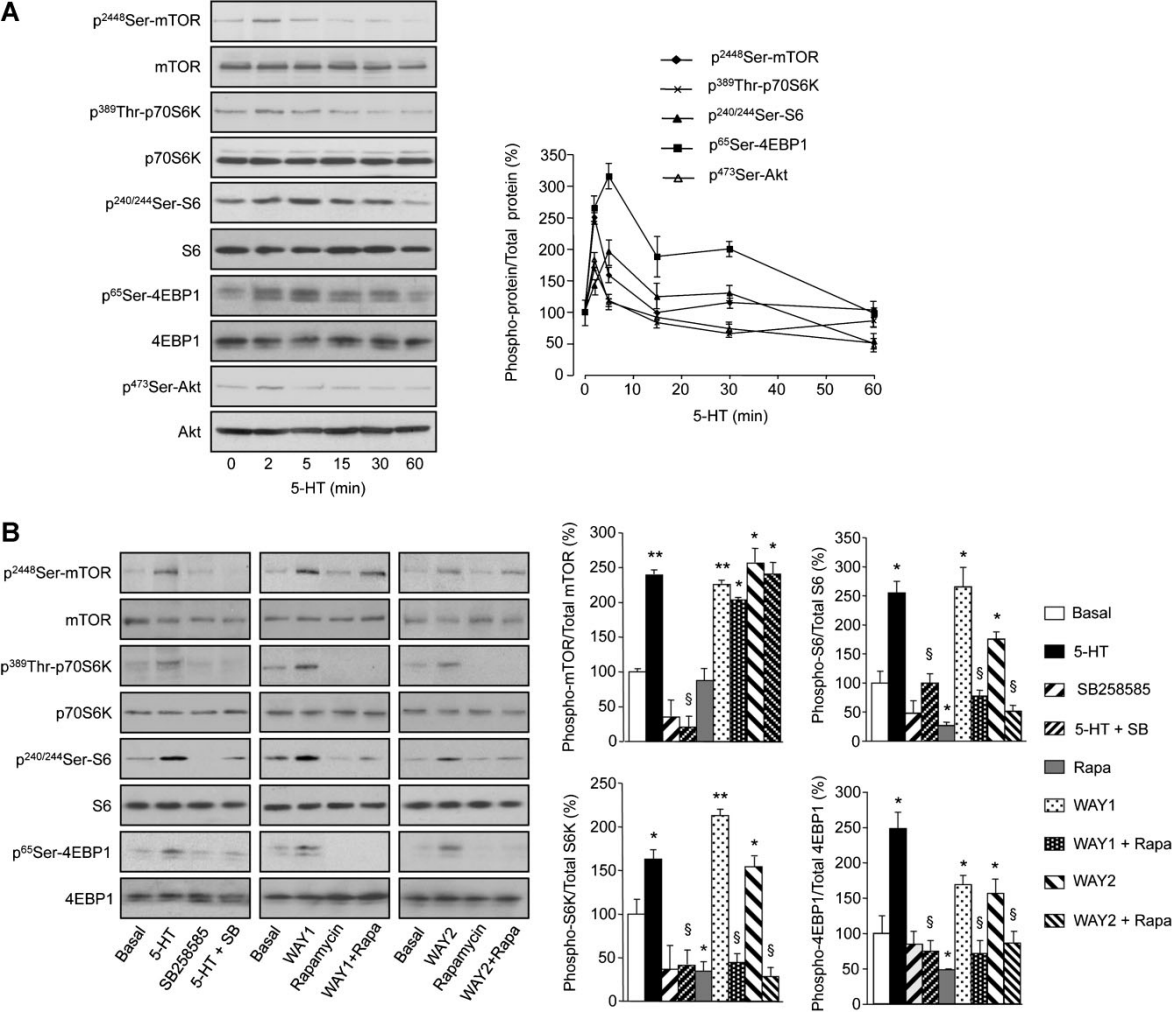
Transient stimulation of mTOR signalling upon 5-HT_6_ receptor activation in HEK-293 cells Representative immunoblots of three independent experiments are shown. Data, expressed as ratios of phosphorylated to total proteins, represent the means ± SEM of values obtained in three independent experiments. HEK-293 cells expressing HA-5-HT_6_ receptor were challenged with 5-HT (1 µM) for the indicated times. mTOR signalling was assessed by sequential immunoblotting with antibodies against phosphorylated mTOR (Ser^2448^), p70S6K (Thr^389^), S6 (Ser^240/244^), 4EBP1 (Ser^65^) and Akt (Ser^473^) and antibodies recognizing the corresponding proteins independently of their phosphorylation state. Immunoreactive signals were quantified by densitometry.Cells were exposed for 2 min to 1 µM of either 5-HT, WAY181187 (WAY1) or WAY208466 (WAY2). They were pretreated with SB258585 (10 µM) or rapamycin (1 µM) for 10 min before agonist exposure (*p* < 0.05, **p < 0.01 *vs.* basal, § *p* < 0.05 *vs.* the corresponding condition in absence of SB258585 or rapamycin, ANOVA followed by Newman–Keuls test).

Exposure of 5-HT_6_ receptor-expressing HEK-293 cells to 5-HT (1 µM) induced a transient (<5 min) increase of mTOR phosphorylation at Ser^2448^, which reflects mTOR activation status, and of its substrate p70 ribosomal S6 kinase (p70S6K) at Thr^389^ (Fig 2A). 5-HT induced a more sustained (≍30 min) increase in the phosphorylation of two other downstream substrates of mTOR, 4EBP1 (at Ser^65^) and S6 (at Ser^240/244^). The 5-HT-induced phosphorylation of mTOR and its substrates was prevented by SB258585 (10 µM), a specific 5-HT_6_ receptor antagonist, and was reproduced by WAY181187 and WAY208466 (1 µM each, Fig 2B). 5-HT_6_ receptor-elicited mTOR signalling was comparable to that induced by insulin-like growth factor 1 or epidermal growth factor (Supporting Information Fig S3) and was strongly decreased in cells expressing 5-HT_6_Δ49Ct receptor, which did not co-immunoprecipitate with mTOR but fully activated cAMP production compared with cells expressing the wild-type receptor (Supporting Information Fig S2).

**Figure 3:**
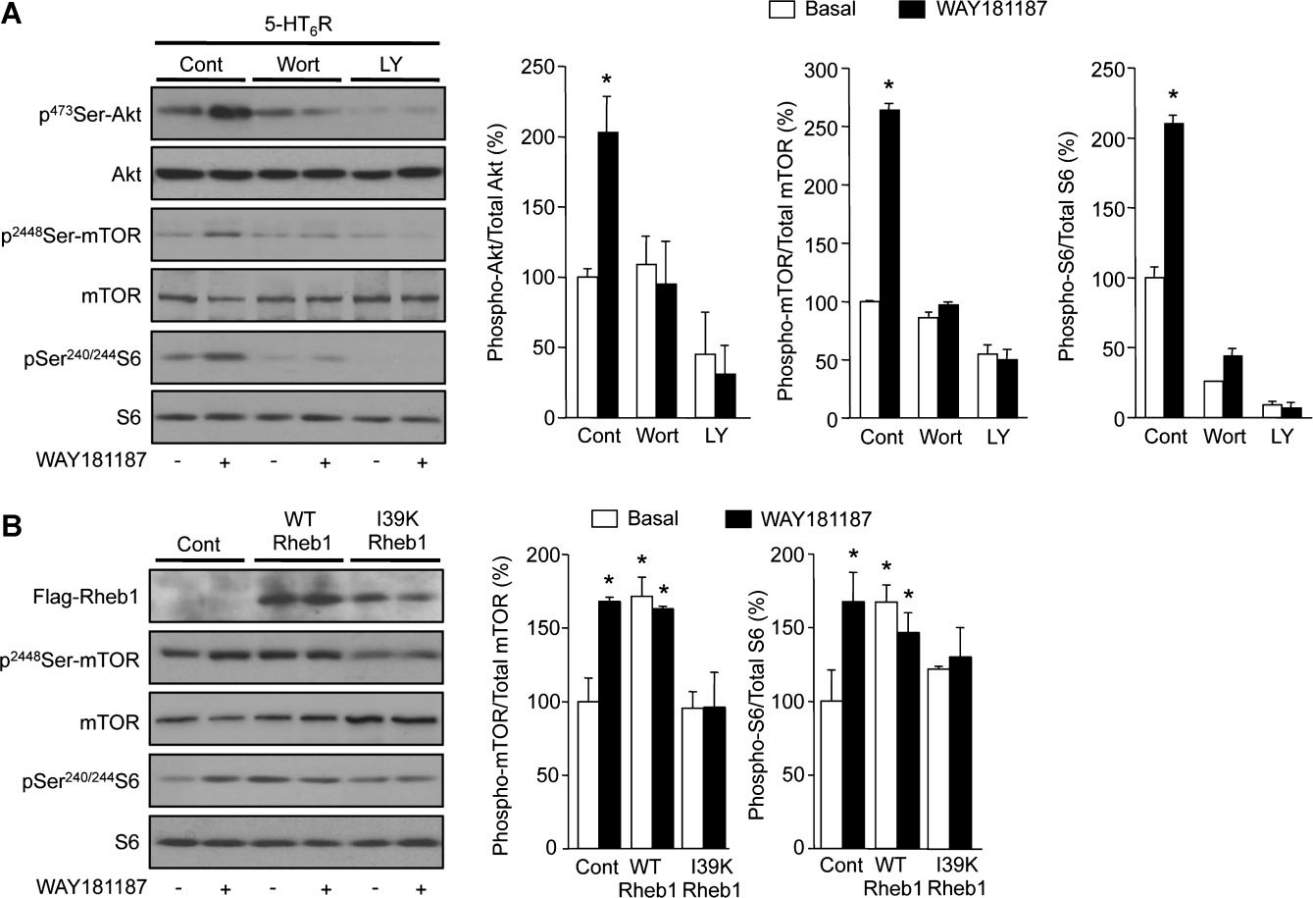
Involvement of the PI3K/Akt/Rheb pathway in 5-HT_6_ receptor-mediated mTOR activation in HEK-293 cells Representative immunoblots of three independent experiments are shown. Data, expressed as ratios of phosphorylated to total proteins, represent the means ± SEM of values obtained in three independent experiments. Cells were pretreated with either vehicle (Cont) or wortmannin (Wort, 100 nM) or LY294002 (20 µM) before a 2-min challenge with WAY181187 (1 µM) (*p* < 0.05 *vs.* basal).Cells, co-transfected with the plasmid encoding HA-5-HT_6_ receptor and either empty vector (Cont) or plasmids encoding Flag-tagged wild-type Rheb1 or dominant-negative Rheb1 (I39K), were challenged for 2 min with WAY181187 (1 µM) (*p* < 0.05 *vs.* basal).

In line with activation of mTORC1 by 5-HT_6_ receptors, phosphorylation of p70S6K (Thr^389^), 4EBP1 (Ser^65^) and S6 (Ser^240/244^) by WAY181187 were prevented by rapamycin, a specific mTORC1 inhibitor, whereas, as expected, phosphorylation of mTOR (Ser^2448^) was unaffected (Fig 2B). Moreover, and consistent with the 5-HT-elicited transient activation of Akt (assessed by phosphorylation at Ser^473^, Fig 2A) that paralleled mTOR phosphorylation, mTOR activation was dependent on the canonical class I phosphatidyl inositol 3-kinase (PI3K)/Akt signalling: phosphorylation of both mTOR (Ser^2448^) and S6 (Ser^240/244^) was strongly reduced in cells pretreated with the PI3K inhibitors wortmannin (100 nM) or LY294002 (20 µM, Fig 3A). Activated Akt can phosphorylate tuberin (TSC2) (Dan et al, 2002; Inoki et al, 2002; Manning et al, 2002), which together with hamartin (TSC1) constitutes the tuberous complex (TSC1/2). TSC1/2 is a GAP for Rheb (Ras homolog enriched in brain), a major upstream activator of mTORC1 (Garami et al, 2003; Inoki et al, 2003; Tee et al, 2003). Phosphorylation of TSC2 by Akt inhibits GAP activity of the complex, resulting in increased levels of Rheb-GTP that in turn stimulates mTOR (Garami et al, 2003; Inoki et al, 2003; Tee et al, 2003). Rheb was not identified by mass spectrometry in the 5-HT_6_ receptor complex purified from HEK-293 cells (Fig 1B and Supporting Information Table S1), likely due to its low expression in these cells. Nonetheless, GST pull-down followed by Western blotting showed recruitment of Rheb from mice brain by the 5-HT_6_ receptor C-terminus (Supporting Information Fig S2C). Moreover, Rheb contributed to 5-HT_6_ receptor-dependent mTOR signalling in HEK-293 cells: both mTOR (S^2448^) and S6 (S^240/244^) phosphorylations elicited by WAY181187 were prevented by expression of a Rheb-dominant-negative mutant (I39K, Fig 3B). In contrast, over-expression of wild-type Rheb itself increased phosphorylation of these residues, which was not further enhanced by WAY181187 exposure (Fig 3B).

### 5-HT_6_ receptors activate mTOR signalling in the prefrontal cortex and the striatum

**Figure 4:**
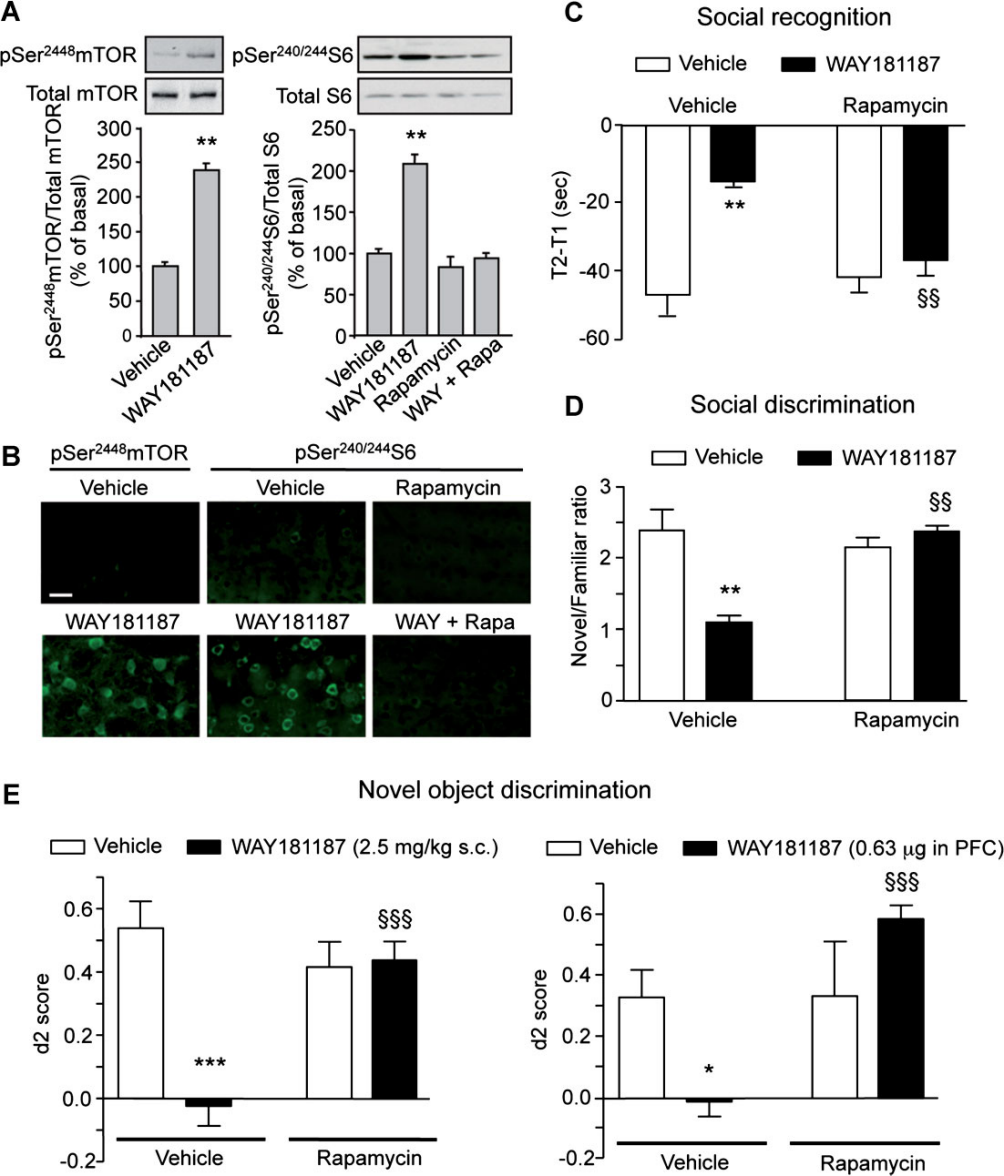
Enhanced mTOR signalling in PFC mediates the cognitive impairments induced by administration of a 5-HT_6_ receptor agonist **A.** Western blot analysis of phosphorylation of mTOR (S^2448^) and S6 (S^240/244^) in PFC of mice treated with vehicle or WAY181187 (10 mg/kg, i.p., 30 min). Rapamycin (10 mg/kg, i.p.) was administered 15 min before WAY181187. Data, expressed as ratios of phosphorylated mTOR or S6 to total mTOR or S6, are means ± SEM (*n* = 4). **p < 0.01, *versus* vehicle.**B.** Immunofluorescent detection of cells positive for phospho-Ser^2448^-mTOR and phospho-Ser^240/244^-S6 in the PFC. Scale bar: 40 µm.**C,D.** Inhibition by rapamycin of alteration of social cognition in rats treated with WAY181187. Data are means ± SEMs and represent the difference in duration of social interaction between the two sessions (T2-T1, *n* = 6 rats per group, **C**) and the ratios of time spent investigating the novel juvenile rat to time spent investigating the familiar one during the choice trial (n = 7–8, **D**). **p < 0.01 *versus* vehicle/vehicle, §§ *p* < 0.01 *versus* vehicle/WAY181187.**E.** Reversal by rapamycin (10 mg/kg s.c.) of the deficit of NOD (assessed by the d2 score) induced by systemic (2.5 mg/kg i.p., left panel) and local (0.63 µg per side in PFC, right panel) administration of WAY181187. Data are means ± SEMs (*n* = 6–12). *p < 0.05, ***p < 0.001 *versus* vehicle/vehicle, §§§ *p* < 0.001 *versus* vehicle/WAY181187.

Corroborating observations in HEK-293 cells, systemic administration of WAY181187 (10 mg/kg i.p.) to mice increased levels of phosphorylated mTOR (Ser^2448^) and S6 (Ser^240/244^), as assessed by Western blotting analysis, in the prefrontal cortex (PFC), a cerebral region involved in the modulation of social cognition by 5-HT_6_ receptor ligands (Loiseau et al, 2008; Fig 4A). This result was confirmed by immunofluorescence staining, which revealed a strong increase in the number of phospho-Ser^2448^-mTOR and phospho-Ser^240/244^-S6-positive cells in the PFC of animals treated with WAY181187 (Fig 4B) or WAY208466 (10 mg/kg each, Supporting Information Fig S4) *versus* vehicle-treated animals. Phosphorylation of S6 by WAY181187 and WAY208466 was abolished by SB258585 (10 mg/kg, i.p., delivered 15 min before the agonists), which itself did not significantly affect S6 phosphorylation (Supporting Information Fig S4). Further supporting engagement of the mTOR pathway by 5-HT_6_ receptor activation, phosphorylation of S6 by WAY181187 was likewise prevented by acute rapamycin administration (10 mg/kg, i.p., 15 min before agonist injections, Fig 4A and B). Notably, this systemic administration of rapamycin was followed by its long-lasting (>2 h after injection) accumulation within the brain (Supporting Information Fig S5). Consistent with data on receptor localization in PFC (Codony et al, 2011; Woolley et al, 2004), phospho-S6 immunoreactivity was detected in both GABAergic neurons [mostly dopamine and cAMP-regulated phosphoprotein (DARPP32)-negative] and non-GABAergic neurons (Supporting Information Figs S6 and S7). Robust 5-HT_6_ receptor-mediated activation of mTOR signalling was also detected in striatum (Supporting Information Fig S4), the brain structure expressing the highest density of 5-HT_6_ receptors. Therein, more than 90% of phospho-Ser^240/244^-S6-stained cells were positive for DARPP32 and correspond to GABAergic medium-sized spiny neurons (Supporting Information Fig S6).

### Rapamycin administration prevents deficits of social cognition and novel object discrimination induced by the 5-HT_6_ receptor agonist WAY181187

As previously shown (Loiseau et al, 2008) and consistent with a role of 5-HT_6_ receptors in social cognition (*i.e.* the complex set of processes used to acquire, interpret and store information about a subject's social environment, including the identity, intentions and behaviour of others), systemic administration of WAY181187 (10 mg/kg, i.p.) to adult rats significantly impaired social recognition in a procedure where a juvenile rat was presented to an adult for two consecutive 5 min sessions (Fig 4C). This action of WAY181187, which was abrogated by SB258585 (Loiseau et al, 2008), was prevented by rapamycin (10 mg/kg, i.p., administered 15 min before the agonist), which alone did not affect social recognition (Fig 4C). Rapamycin, like SB258585 (Supporting Information Fig S8A), also abrogated the deficit induced by WAY181187 in the social novelty discrimination procedure (Fig 4D). Consistent with a specific role of mTOR in the cognitive impairment induced by 5-HT_6_ receptor activation, rapamycin did not block the deficit in social recognition induced by the muscarinic receptor antagonist scopolamine (1.25 mg/kg s.c., Supporting Information Fig S9). In agreement with the documented improvement of recognition memory by 5-HT_6_ receptor antagonism (Woolley et al, 2004), systemic administration of WAY181187 to rats also impaired visual episodic-like memory in a non-spatial novel object discrimination (NOD) procedure, which was reversed by both rapamycin (Fig 4E) and SB258585 (Supporting Information Fig S8B). Neither WAY181187, SB258585 nor rapamycin, alone or in combination, significantly altered total object exploration in this task (Supporting Information Fig S8B and S10), confirming their specific action on cognition. As previously shown for social cognition (Loiseau et al, 2008), local delivery of WAY181187 (bilaterally, 0.63 µg/side) into the PFC also impaired NOD, an effect prevented by peripheral rapamycin administration (Fig 4E). Collectively, these results indicate that mTOR signalling mediates the cognitive impairments induced by activation of 5-HT_6_ receptors in PFC in two cognitive paradigms with clear relevance to the impairment of social cognition, attention and episodic memory observed in schizophrenia.

### Rapamycin administration prevents cognitive deficits in two developmental models of schizophrenia, neonatal phencyclidine treatment and post-weaning rearing in isolation

**Figure 5:**
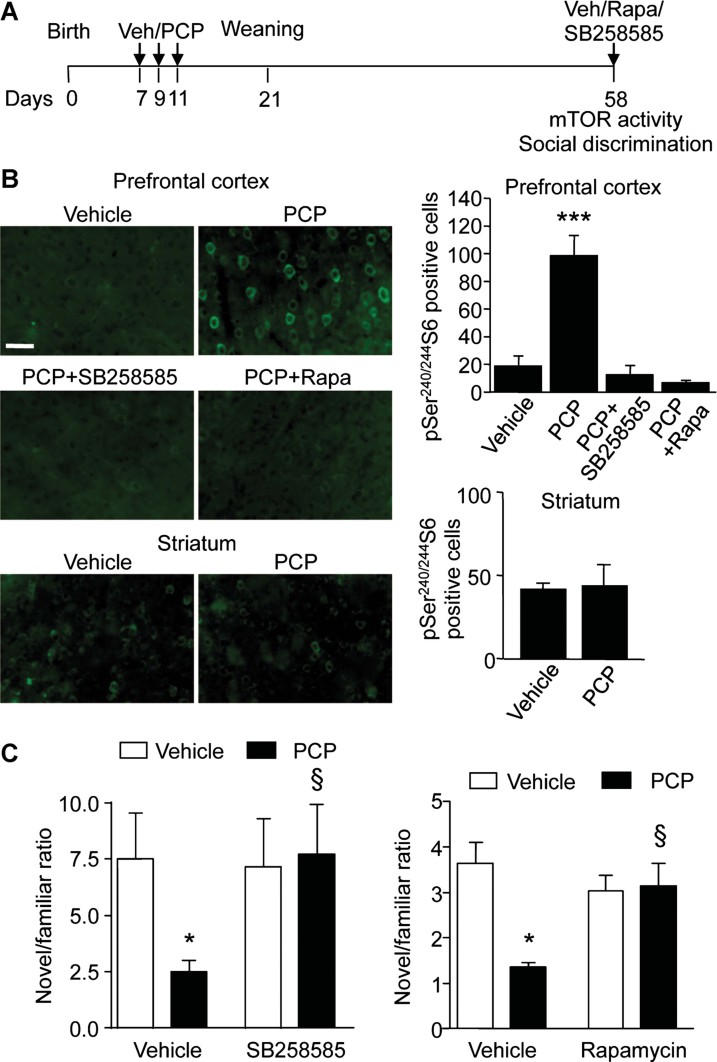
5-HT_6_ receptor-elicited mTOR signalling in PFC underlies impairment of social discrimination in rats treated with phencyclidine during the neonatal period Schema of the experimental paradigm used.Immunofluorescent detection and quantification of cells positive for phospho-Ser^240/244^-S6 in the PFC and the striatum of vehicle- or PCP- (10 mg/kg, s.c.) treated rats that received either acute (30 min before brain perfusion) injection of SB258585 (2.5 mg/kg i.p.), rapamycin (10 mg/kg i.p.) or vehicle. Error bars represent SEM (n = 4). Quantification was performed on 224 µm × 168 µm images. ***p < 0.001 *versus* vehicle-treated rats. Scale bar: 40 µm.Vehicle- or PCP-treated rats were treated with SB258585 (2.5 mg/kg i.p.), rapamycin (10 mg/kg i.p.) or vehicle 30 min before the first session of the trial. Data, expressed as ratios of time spent investigating the novel juvenile rat to time spent investigating the familiar one during the 5-min choice trial, are means ± SEM obtained in *n* = 7–8 rats per group. *p < 0.05 *versus* vehicle/vehicle, § *p* < 0.05 *versus* PCP/vehicle.

We next examined the role of 5-HT_6_ receptor-mediated mTOR signalling in rats that had received repeated injections of the psychotomimetic phencyclidine (PCP) during the neonatal period (Fig 5A), a treatment that provokes cognitive and other behavioural changes characteristic of schizophrenia in the mature animal (Jones et al, 2011). A marked increase in phospho-S6-positive neurons was detected in PFC of adult rats treated as neonates with PCP compared with vehicle-treated animals (Fig 5B). Conversely, underpinning the specificity of this change, neonatal PCP treatment did not increase S6-phosphorylation in striatum (Fig 5B). S6 phosphorylation in PFC of PCP-treated rats was abolished by acute (30 min, i.p.) treatment with rapamycin (10 mg/kg) or SB258585 (2.5 mg/kg). These findings suggest that neonatal treatment with PCP leads to delayed increase in 5-HT_6_ receptor-mediated mTOR signalling specifically in the PFC, a structure critically implicated in the cognitive deficits relevant to those seen in schizophrenia. Correspondingly, the deficit in social discrimination observed in PCP-treated rats was abolished by either SB258585 or rapamycin (Fig 5C).

**Figure 6:**
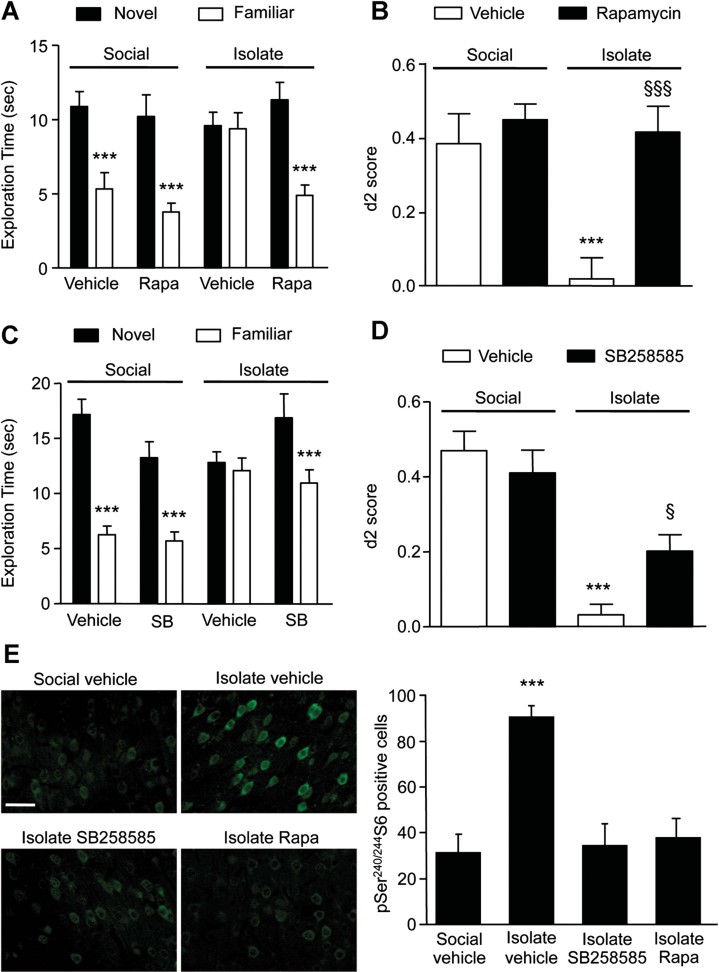
5-HT_6_ receptor-elicited mTOR signalling in PFC underlies deficit in NOD in rats housed in isolation Rats were weaned on post-natal day (PND) 24 and pups from each litter divided and housed in either groups (3–4 per cage) or in social isolation for 6 weeks. Both groups were injected with either vehicle or rapamycin or SB258585 (on PND 66) 30 min prior to the familiarization trial. The interval between familiarization and choice trials was 2 h. Reversal by rapamycin (10 mg/kg i.p.) of isolation-induced impairment in NOD during the choice trial. Data are means ± SEMs (*n* = 9–10 rats per group). Isolated rats were significantly impaired in the NOD task (*p* < 0.05). ***p < 0.001 *versus* novel object in the same group.Reversal by rapamycin of isolation-induced impairment in NOD as measured by the d2 score. ***p < 0.001 *versus* social/vehicle group. §§§ *p* < 0.001 *versus* isolate/vehicle group.Reversal by SB258585 (10 mg/kg i.p.) of the impairment in NOD produced by isolation rearing. Isolated rats showed a significant impairment in object discrimination (*p* < 0.001). ***p < 0.001 *versus* novel object in same treatment group.Reversal by SB258585 of the isolation-induced impairment in NOD, as measured by the d2 score. ***p < 0.001 *versus* social/vehicle group. § *p* < 0.05 *versus* isolate/vehicle group.Immunofluorescent detection and quantification of cells positive for phospho-Ser^240/244^-S6 in the PFC of rats housed in groups or in isolate, injected with either vehicle or rapamycin or SB258585. Quantification was performed on 448 µm × 335 µm images. ***p < 0.001 *versus* social/vehicle group. Scale bar: 40 µm.

Systemic administration of rapamycin (10 mg/kg) or SB258585 (10 mg/kg) also reversed the NOD deficit produced by housing rats in social isolation from the day of weaning (Fig 6A–D), an alternative neurodevelopmental model of schizophrenia (Fone & Porkess, 2008; Marsden et al, 2011). As observed in PCP-treated rats, we found an increase in mTOR signalling (assessed by S6 phosphorylation) in the PFC of isolated rats compared with group-housed animals, a difference abolished by administration of rapamycin or SB258585 (Fig 6E).

## DISCUSSION

In the present study, we performed an unbiased proteomic analysis of the 5-HT_6_ receptor complex in an effort to identify novel signalling mechanisms underlying its control of cognition. Six of the 28 proteins found to reproducibly interact with 5-HT_6_ receptors expressed in HEK-293 cells are proteins of the mTOR pathway. These include two core components of mTORC1, mTOR itself and Raptor, which constitutively binds to mTOR and is involved in recruiting substrates for phosphorylation by the kinase domain of mTOR (Wang & Proud, 2011). Physical association of mTOR with native 5-HT_6_ receptors expressed in mice brain was further validated by co-immunoprecipitation, providing strong evidence that 5-HT_6_ receptor and mTORC1 form a complex *in vivo*. To our knowledge, this is the first demonstration of a physical interaction of mTORC1 with a GPCR.

Although several proteins of the mTOR pathway [*e.g.* GβL, the third core component of mTORC1 (Wang & Proud, 2011)] were not identified in our proteomic screen, the possibility that additional partners involved in mTOR signalling interact with 5-HT_6_ receptors should not be discounted. Their absence in the identified interactome might reflect a transient interaction with the receptor complex, loss of interaction during receptor solubilization and/or insufficient expression in the heterologous system used. For instance, pull-downs performed from brain tissue, which express high levels of Rheb (presumably higher than in HEK-293 cells), clearly identified Rheb as a 5-HT_6_ receptor interacting protein, whereas Rheb was not identified by mass spectrometry in the initial proteomic screen.

The precise architecture of the complex formed by 5-HT_6_ receptors and mTORC1 also remains to be established. Mass spectrometry analyses identified many more peptides in the mTOR sequence (42 unique peptides corresponding to 20.2% sequence coverage) than in the Raptor sequence (five unique peptides corresponding to 4.7% sequence coverage). This suggests that 5-HT_6_ receptors recruit larger amounts of mTOR than Raptor, since it is unlikely that these values only reflect a difference in the number of suitably ionizable tryptic peptides in both proteins. Accordingly, mTOR might be a direct partner of the 5-HT_6_ receptor, whereas Raptor might be recruited indirectly via mTOR. In addition, we showed that the 49 C-terminal amino acids of the 5-HT_6_ receptor were necessary for interacting with mTOR but that the receptor C-terminal domain alone was not capable of recruiting mTOR. This suggests that several domains of the receptor, including both its extreme C-terminus and residues located upstream to its C-terminal domain, contribute to receptor-mTOR interaction.

The remarkable enrichment in proteins of the mTOR pathway in the 5-HT_6_ receptor interactome was complemented by observations of its engagement by the receptor both in transfected HEK-293 cells and *in vivo*, providing the first evidence of a functional link between serotonergic transmission and mTOR signalling. The PI3K/Akt/Rheb pathway classically involved in mTOR activation by insulin and other growth factors (Swiech et al, 2008; Wang & Proud, 2011; Zhou & Huang, 2010) was likewise implicated in the 5-HT_6_ receptor-elicited mTOR signalling in HEK-293 cells. Moreover, the deletion of the 49 C-terminal residues of receptor, which prevented its interaction with mTOR, strongly reduced its ability to activate mTOR, without affecting its coupling to Gαs. This suggested that 5-HT_6_ receptor-elicited mTOR signalling was critically dependent of their physical association with mTOR. The lack of a unique, well-localized mTOR-binding motif in the 5-HT_6_ receptor sequence precluded the use of an ‘interfering peptide’ in order to disrupt its interaction with mTOR and to further explore the functional impact of this coupling. In any event, it is possible that constitutive physical interaction of 5-HT_6_ receptors with mTOR facilitates its engagement, via PI3K and Akt, upon receptor stimulation. It may also help in confining 5-HT_6_ receptor-elicited mTOR signalling to specific cellular domains, thereby permitting local regulation of mTOR down-stream targets by 5-HT_6_ receptors.

Peripheral administration of a 5-HT_6_ receptor agonist markedly activated mTOR in striatal medium spiny neurons, which express the highest density of 5-HT_6_ receptors in the brain (Codony et al, 2011; Woolley et al, 2004). Activation of mTOR in striatum via Rhes, a striatal-specific small G protein, is known to mediate l-DOPA-induced dyskinesia (Santini et al, 2009; Subramaniam et al, 2011). It has also been proposed that blockade of striatal mTOR signalling caused by the sequestration of Rhes by mutant Huntingtin might underlie the pronounced atrophy of the striatum in Huntington disease (Subramaniam & Snyder, 2011). Whether these processes are regulated by 5-HT_6_ receptors remains to be established.

Administration of a 5-HT_6_ receptor agonist also stimulated mTOR signalling in neurons of PFC, the cerebral structure involved in their control of social cognition (Loiseau et al, 2008). Neurons exhibiting enhanced mTOR signalling upon 5-HT_6_ receptor activation included GABAergic neurons consistent with data on receptor localization, which indicated that almost 20% of 5-HT_6_-immunoreactive neurons in cerebral cortex were GABAergic (Codony et al, 2011).

The mTOR pathway plays a crucial role in neurodevelopmental processes, including cell proliferation, synaptogenesis and growth of dendrites and axons and its perturbation has been implicated in the cognitive deficits of two rare genetic forms of autism spectrum disorders, tuberous sclerosis and fragile X syndrome (Auerbach et al, 2011; Ehninger et al, 2008; Ehninger & Silva, 2011; Swiech et al, 2008). These findings, together with our observations that 5-HT_6_ receptors engage mTOR in neurons of the PFC, a structure critical for numerous cognitive functions, encouraged us to explore the role of prefrontal mTOR in cognitive impairments induced by 5-HT_6_ agonists. We found that systemic administration of the mTOR inhibitor rapamycin prevented cognitive deficits induced by a 5-HT_6_ agonist (also administered at the periphery) in models of social cognition (primarily involving olfactory cues) and NOD (visual cues). Further supporting a role of prefrontocortical mTOR activation, rapamycin administration also abolished the deficit in NOD induced by a local injection of a 5-HT_6_ agonist in the PFC.

We then reasoned that enhanced mTOR signalling in the PFC, under the control of 5-HT_6_ receptors, might underlie cognitive deficits of schizophrenia. Consistent with this hypothesis, we observed an enhanced mTOR signalling (blocked by a 5-HT_6_ antagonist) in PFC of adult rats treated with PCP at a neonatal stage or housed in isolation after weaning from the dam. These two well-characterized and complementary developmental models of schizophrenia reproduce in adulthood several features of schizophrenia such as deficits in social cognition (neonatal PCP administration) and episodic memory (isolation rearing; Jones et al, 2011; Marsden et al, 2011). These cognitive deficits were abolished by an acute administration of rapamycin, mirroring the effects of 5-HT_6_ antagonists. In both models, the increased mTOR signalling was specifically detected in PFC, and not in striatum, contrasting with the effects induced by agonist treatment. These findings are consistent with the dysfunction of prefrontal GABAergic transmission in schizophrenia, which is associated with gene expression abnormalities in a subclass (paravalbumin-positive) of PFC GABAergic interneurons (Hashimoto et al, 2003). They support the notion that certain cognitive defects in schizophrenia reflect a disruption of PFC function and connectivity, itself related to anomalous activity of GABAergic interneurons and a loss of their synchronized modulation of pyramidal neurons (Lewis & Gonzalez-Burgos, 2008). Alterations in network connectivity might ultimately be related to a deregulation of mTOR signalling in the PFC, occurring at a critical period in post-natal brain development and persisting into adulthood.

**Figure 7:**
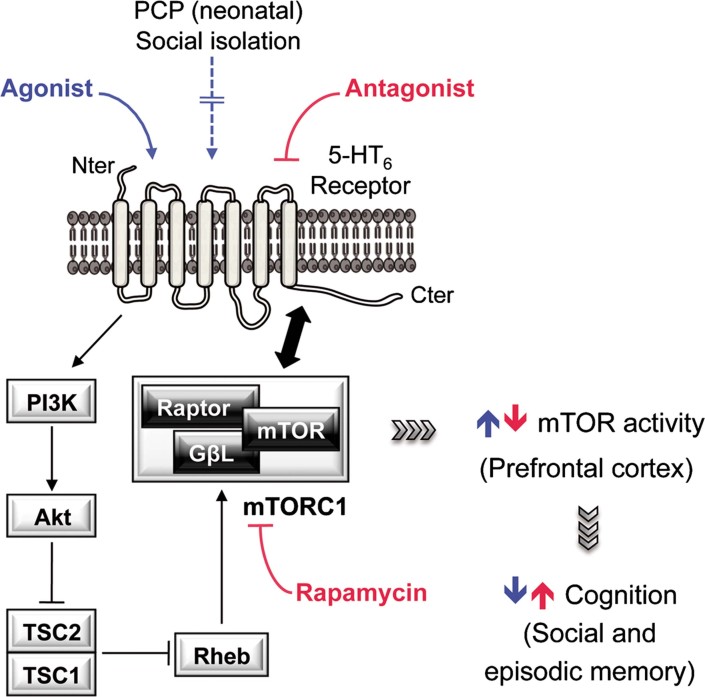
5-HT_6_ receptors recruit the mTOR complex 1 to compromise cognition in rodent developmental models of schizophrenia The 5-HT_6_ receptor physically interacts with proteins of the mTOR pathway, including mTOR itself and Raptor, which with GβL constitute the rapamycin-sensitive mTOR complex 1 (mTORC1). This interaction, together with the canonical class I PI3K/Akt/Rheb pathway, contributes to 5-HT_6_ receptor-elicited mTOR signalling, which underlies the cognitive deficits observed in two developmental models of schizophrenia, neonatal treatment with phencyclidine and post-weaning social isolation.

In conclusion, the present observations demonstrate that 5-HT_6_ receptors recruit and activate mTOR to compromise cognition both in pharmacological paradigms and in developmental models of schizophrenia (Fig 7). Accordingly, activation of mTOR may fulfil a broader role in the cognitive impairment of CNS disorders than hitherto appreciated. Indeed, our findings encourage an extension of clinical trials of mTOR inhibitors from patients presenting genetic forms of autism-related disorder, like tuberous sclerosis (Auerbach et al, 2011; Ehninger et al, 2008; Ehninger & Silva, 2011), to more numerous populations of schizophrenic patients. They reciprocally suggest that 5-HT_6_ antagonists might profitably be evaluated in autism. Irrespective of the outcome, the present work may help to resolve and to inter-link two persistent conundrums: namely, the molecular substrates mediating the detrimental influence of 5-HT_6_ receptors on cognition and the cellular events accounting for cognitive deficits in schizophrenia.

## MATERIALS AND METHODS

### Plasmids, chemicals and antibodies

The human 5-HT_6_ receptor cDNA, purchased from IMAGE (30915608), was subcloned in pRK5 vector into the BsrGI and HindIII sites and fused amino-terminally to a HA tag. The 5-HT_6_Δ49Ct receptor construct was obtained by inserting a stop codon after glutamine 391 in the HA-5-HT_6_ receptor sequence. The HA-5-HT_7_ receptor cDNA was kindly provided by Dr E. Ponimaskin (Göttingen, Germany), Flag-Rheb1 (WT and I39K mutant) cDNAs by Dr J. Avruch (Boston, MA). The cDNA encoding 5-HT_6_ receptor C-terminus (last 120 residues) was amplified by PCR and subcloned in the pGEX-3X vector (GE Healthcare) using the *Bam*HI and *Eco*RI restriction sites.

SB258585 (4-iodo-*N*-[4-methoxy-3-(4-methyl-piperazin-1-yl)-phenyl]benzenesulphonamide) was obtained from Tocris. WAY181187 (2-(1-(6-chloroimidazo[2,1-b]thiazol-5-ylsulfonyl)-1H-indol-3-yl)ethanamine)) and WAY208466 (*N*-[2-[3-(3-fluorophenylsulfonyl)-1H-pyrrolo[2,3-b]pyridin-1-yl]ethyl]-*N*,*N*-dimethylamine) were synthesized by Gilbert Lavielle (IDRS, France). 5-HT (creatinine sulfate), scopolamine and HA peptide were purchased from Sigma–Aldrich, rapamycin from LC Laboratories, wortmannin and LY294002 from Calbiochem. The agarose-conjugated anti-HA antibody and the mouse anti-Flag antibody were obtained from Sigma–Aldrich, the rabbit anti-HA antibody from Invitrogen, the mouse anti-NeuN antibody from Millipore, the mouse anti GABA antibody from Chemicon, the rabbit anti-Neurofibromin 1 antibody from Santa-Cruz Biotechnology and the rabbit anti 5-HT_6_ receptor antibody from GeneTex, Inc. The mouse monoclonal anti-DARPP32 antibody was described elsewhere (Snyder et al, 1992). Rabbit polyclonal antibodies against phospho-Ser^2448^-mTOR, phospho-Ser^240/244^-S6, phospho-Thr^389^-P70S6K, phospho-Ser^65^-4EBP1, phospho-Ser^473^-Akt, total mTOR, S6, P70S6K, 4EBP1, Rheb, Raptor and Akt were from Cell Signaling Technology.

### Cell culture and transfection

HEK-293 cells, grown in Dulbecco's modified Eagle's medium (DMEM) supplemented with 10% dialysed, heat-inactivated foetal calf serum and antibiotics, were transfected using polyethyleneimine (PEI, Sigma–Aldrich) and used 24 h after transfection.

### Purification and identification of 5-HT_6_ receptor-interacting proteins by mass spectrometry

HEK-293 cells expressing HA-5-HT_6_ receptor were lysed in a buffer containing HEPES 20 mM, pH 7.4, 150 mM NaCl, 1% NP40, 10% glycerol, 4 mg/ml dodecylmaltoside and a protease inhibitor cocktail (Roche), for 1 h at 4°C. Samples were centrifuged at 15,000 × *g* for 30 min at 4 °C. Solubilized proteins (10 mg per condition) were immunoprecipitated with the agarose-conjugated HA antibody (100 µl per condition). Control immunoprecipitations were performed in the presence of HA peptide (100 µM). Immunoprecipitated proteins were eluted in Laemmli sample buffer, separated by SDS–PAGE and stained with Page Blue Stain (Fermentas). Gel lanes were cut into 20 gel pieces and proteins digested in-gel using trypsin (Gold, Promega). Generated peptides were analysed online by nano-flow HPLC-nanoelectrospray ionization using a LTQ Orbitrap XL mass spectrometer (Thermo Fisher Scientific) coupled with an Ultimate 3000 HPLC (Dionex). Desalting and pre-concentration of samples were performed on-line on a Pepmap® precolumn (0.3 mm × 10 mm, Dionex). A gradient consisting of 0–40% A in 30 min, 80% B in 15 min (A = 0.1% formic acid, 2% acetonitrile in water; B = 0.1% formic acid in acetonitrile) at 300 nl/min was used to elute peptides from the capillary (0.075 mm × 150 mm) reverse-phase column (Pepmap®). LC-MS/MS experiments comprised cycles of five events; an MS^1^ scan with Orbitrap mass analysis at 30,000 resolution followed by CID of the five most abundant precursors. Fragment ions generated by CID were detected in the linear trap. Normalized collision energy of 35 eV and activation time of 30 ms were used for CID. All Spectra were recorded under positive ion mode using the Xcalibur 2.0.7 software (Thermo Fisher Scientific). The mass scanning range (*m*/*z*) was 400–2000 and the capillary temperature was 200°C. Source parameters were adjusted as follows: ion spray voltage, 2.40 kV; capillary voltage, 40 V and tube lens, 120 V. Spectra were acquired with the instrument operating in the information-dependent acquisition mode throughout the HPLC gradient.

All MS/MS spectra were searched against the Homo sapiens entries of either SwissProt or TrEMBL databases (http://www.uniprot.org/) by using the Mascot v 2.2 algorithm (Matrix Science, http://www.matrixscience.com/) with trypsin enzyme specificity and one trypsin missed cleavage. Methionine oxidation was set as variable modification for searches. A peptide mass tolerance of 5 ppm and a fragment mass tolerance of 0.5 Da were allowed for identification. Management and validation of mass spectrometry data, allowing discrimination of specific HA-tagged 5-HT_6_ receptor partners, were performed using the myProMS v2.3. Web server (Poullet et al, 2007). Experiments were repeated four times to assess biological reproducibility. Only proteins identified with two or more peptides (threshold Mascot scores given corresponding to *p* < 0.01 for two peptides/protein and *p* < 0.001 for three peptides or more/protein, respectively) in each replicate and not detected in control immunoprecipitations were considered as potential partners of 5-HT_6_ receptor.

GO annotations and functional enrichment in the 5-HT_6_ receptor complex were analysed using BINGO (version 2.44), a Cytoscape plugin assessing overrepresentation of Gene Ontology categories in biological networks (Maere et al, 2005). The list of proteins interacting specifically with the 5-HT_6_ receptor was used as the input set and the whole annotation as the reference set. The *p*-value was calculated with the hypergeometric test. For multi-testing correction, we applied the Benjamini & Hochberg False Discovery Rate (FDR) correction with a level of significance of 0.05. To minimize the impact of multi-testing issues, we used the GOSlim ontologies.

### GST pull-down

Ten micrograms of fusion proteins (produced in BL21 cells), immobilized onto glutathione sepharose beads (GE Healthcare), were incubated overnight at 4°C with 5 mg of solubilized proteins from mice brain. After five washes with 0.5 M NaCl, retained proteins were eluted from the beads and analysed by Western blotting.

### Western blotting

Equal amounts of protein (30 µg) for each sample were resolved onto 10% polyacrylamide gels. Proteins were transferred to Hybond C nitrocellulose membranes (GE Healthcare). Membranes were immunoblotted with primary antibodies (anti phospho-Ser^240/244^-S6, phospho-Ser^473^-Akt, total S6 and total Akt, Raptor and Neurofibromin 1, 1:1000 dilution; anti phospho-Ser^2448^-mTOR, phospho-Thr^389^-P70S6K, phospho-Ser^65^-4EBP1, total mTOR, P70S6K, 4EBP1, Rheb, 1:500 dilution; anti HA and Flag, 1:400 dilution) and then with either anti-mouse or anti-rabbit horseradish peroxidase-conjugated secondary antibodies (1:3000, GE Healthcare). Immunoreactivity was detected with an enhanced chemiluminescence method (ECL™ plus detection reagent, GE Healthcare) and immunoreactive bands were quantified by densitometry using the ImageJ software. In protein phosphorylation analyses, the amount of each phosphoprotein was normalized to the amount of the corresponding total protein detected in the sample. Data were analysed using the GraphPad Prism software (v. 4.0b) and statistical significance determined by one-way ANOVA followed by Newman–Keuls test.

### Drug administration

Experiments were carried out on male Swiss mice (30–35 g) or Wistar rats (220–240 g, Janvier) under standard laboratory conditions and conformed to European ethics standards (86/609-EEC) and to decrees of the French National Ethics Committee (No 87/848) for the care and use of laboratory animals. Studies using Lister Hooded rats (Charles River, UK) were performed in compliance with the UK Home Office Animals (Scientific Procedures) 1986 ACT and local University of Nottingham ethical committee approval. For systemic administration of drugs, animals were injected i.p. or s.c. with drugs as indicated in the figure legends. WAY181187, WAY208466, SB258585 and rapamycin were dissolved in 5% DMSO/5% Tween 80. For combined treatments, rapamycin or SB258585 were administered 15–30 min before the 5-HT_6_ agonist. Animals that did not receive drugs were injected with an equivalent amount of vehicle. WAY181187 was also delivered locally [0.63 µg per side in 1 µl vehicle (10% hydroxypropyl-β-cyclodextrine in a CSF)] by means of cannula implanted bilaterally in the PFC (AP: +3.0, L: ±0.7, DV: −2.3), 1 week before the experiments. For administration of phencyclidine at the neonatal stage, rats were received at 3 days of age, grouped in 10 male pups per adult mother. Phencyclidine or vehicle (saline) was administered on post-natal days (PND) 7, 9 and 11. All pups of the same litter received the same treatment. The rats were weaned from their mother at PND 21, after which they were separated at random and housed in mixed-litter groups of four. Measurement of mTOR activation and social novelty discrimination experiments were carried out at 8–10 weeks of age.

### Analysis of *in vivo* mTOR activation

Activation of mTOR was assessed by Western blotting and immunohistochemistry using anti-phosphoSer^2448^-mTOR and anti-phosphoSer^240/244^-S6 antibodies. For Western blotting, mice were decapitated and their heads were immediately frozen in liquid nitrogen (for 12 s). The frozen heads were cut into 210-µm thick slices with a cryostat. Microdiscs (1.4 mm diameter) were punched out bilaterally from the median PFC and homogenized by the addition of a boiling solution of 1% SDS v/v and 1 mM Na^+^-orthovanadate in water, immediate sonication, and incubation at 100°C for 5 min to inactivate phosphatases and proteases. For immunohistochemistry, animals were rapidly anaesthetized with pentobarbital (100 mg/kg i.p., Ceva SA) and perfused transcardially with fixative solution containing 4% w/v paraformaldehyde in 0.1 M sodium phosphate buffer (pH 7.5) containing NaF (100 mM) and Na^+^-orthovanadate (1 mM). Glutaraldehyde (0.5% w/v) was added to the fixative solution in experiments using the anti-GABA antibody. Brains were post-fixed overnight in the same solutions and stored at 4°C. Fifty micrometer-thick sections were cut with a vibratome (Leica) and stored at 4°C in 0.1 M sodium phosphate buffer (PBS), permeabilized with 0.1% Triton X-100 for 20 min and incubated for 48 h at 4°C with the primary antibodies (phospho-Ser^2448^-mTOR, 1:50; phospho-Ser^240/244^ S6, 1:500; DARPP32, 1:1500; NeuN, 1:1000; GABA, 1:500) in PBS containing 0.025% Triton X-100 and 20% goat serum. Sections were then incubated for 1 h with goat Cy3-conjugated anti-mouse antibody (1:500, Jackson Laboratory) and/or goat Alexa Fluor 488-conjugated anti-rabbit antibody (1:1000, Invitrogen) in PBS containing 20% goat serum. Immunofluorescent staining was observed with a Zeiss Axiophot2 microscope equipped with epifluorescence. Images were acquired using the Metamorph software (Molecular Devices) driving a CoolSNAP CCD camera (Photometrics) and quantification of phospho-Ser^240/244^-S6 positive cells was performed on 224 µm × 168 µm or 448 µm × 336 µm images. Statistical significance was determined by one- or two-way ANOVA followed by the Newman–Keuls test. Double-labelled images from regions of interest were obtained using sequential laser scanning confocal microscopy (Leica SP2 and Zeiss LSM).

**The paper explained**

PROBLEM:

Cognitive deficits in schizophrenia seriously compromise quality of life and are poorly controlled by currently available antipsychotic agents, which mainly correct positive symptoms. Complicating the development of improved therapy, cellular events underlying cognitive impairment in schizophrenia are incompletely understood. 5-HT_6_ receptor blockade has emerged as a promising strategy for correcting these cognitive deficits, as 5-HT_6_ antagonists increase mnemonic performance in a broad range of procedures in rodents and preliminary evidence indicates that they favour cognitive processes in human. However, the signalling mechanisms mediating the control of cognition by 5-HT_6_ receptors remain largely unknown, an issue we addressed by a proteomic analysis of the 5-HT_6_ receptor complex.

RESULTS:

We show that 5-HT_6_ receptors physically interact with the rapamycin-sensitive mTOR complex 1 and that 5-HT_6_ receptor activation elicits mTOR signalling in rodent PFC, a brain region critical for numerous cognitive processes. Correspondingly, the mTOR inhibitor rapamycin prevented deficits of social cognition and NOD induced by a 5-HT_6_ agonist in the rat. Moreover, rapamycin, like 5-HT_6_ antagonists, rescued cognitive deficits in two complementary developmental rodent models of schizophrenia that reproduce in adulthood many features of the disease, neonatal treatment with phencyclidine and post-weaning housing in isolation.

IMPACT:

These studies identify a signalling complex physically associated with the 5-HT_6_ receptor that underlies its deleterious influence upon cognition. They suggest a critical role of mTOR activation not only in rare autism-related genetic disorders, such as Fragile X mental retardation syndrome and tuberous sclerosis, as previously suggested, but also in schizophrenia, a more frequent, multi-factorial and debilitating disorder. These findings encourage an extension of clinical trials of mTOR inhibitors from patients presenting genetic forms of autism to populations of schizophrenic patients and reciprocally suggest that 5-HT_6_ antagonists might be evaluated in autism.

### Behavioural studies

The social recognition test (ability of an adult rat to recognize a younger conspecific during the second of two 5-min sessions) was performed using a procedure without an intersession delay, as previously described (Loiseau et al, 2008). For the social novelty discrimination test (ability of an adult rat to differentiate novel and familiar juveniles), one juvenile rat was introduced in the adult cage in a first 30-min session. The same juvenile rat was then presented together with a second novel juvenile to the adult in a second 5-min session. Time of investigation of each juvenile during the second session was recorded and the ratio of time spent in active social investigation of the novel to the time spent in investigating the familiar during the second session was calculated. We used a procedure without an inter-session interval to maximize discrimination performance (ratio ≥ 3 in vehicle-treated group). The specificity of drug effects on social recognition or social discrimination was analysed by two-way ANOVA, followed by either Newman–Keuls test (social recognition) or Dunnett's test (social discrimination).

The NOD test was performed as previously described (Watson et al, 2011). The inter-trial interval between familiarization and choice trials was 5 min, such that vehicle-treated animals were able to discriminate the novel and familiar objects. In experiments evaluating the impact of social isolation, each litter of Lister hooded rats was weaned on PND 24 and approximately half of each litter housed in either groups (3–4 per cage) or social isolation for 6 weeks. Isolated and group-housed rats were then subjected to the NOD test on PND 66. Injections were carried out 30 min prior to the familiarization trial. The interval between familiarization and choice trials was 2 h. Raw data were analysed by ANOVA with the exploration of novel and familiar object as the repeated within-subject factor and the treatment as the between-subject factor. D2 scores (exploration of novel object – exploration of familiar object/total object exploration) were analysed by two-way ANOVA followed by Newman–Keuls test.

## Supplementary material

Detailed facts of importance to specialist readers are published as ”Supporting Information”. Such documents are peer-reviewed, but not copy-edited or typeset. They are made available as submitted by the authors.
