# Supplementary material for: 5-HT6 receptor recruitment of mTOR as a mechanism for perturbed cognition in schizophrenia
Source: EMBO Mol Med. 2012 Oct 2;4(10):1043–56. doi: 10.1002/emmm.201201410 (PMC3491835; doi:10.1002/emmm.201201410)
Supplement: Supplementary file 1 [file emmm0004-1043-SD1.pdf]

EMBO
Molecular Medicine

Manuscript EMM-2012-01410

5-HT_6 Receptor Recruitment of mTOR as a Mechanism for Perturbed Cognition in Schizophrenia

Julie Meffre, Séverine Chaumont-Dubel, Clotilde Mannoury la Cour, Florence Loiseau, David J.G. Watson, Anne Dekeyne, Martial Séveno, Jean-Michel Rivet, Florence Gaven, Paul Déléris, Denis Hervé, Kevin C.F. Fone, Joël Bockaert, Mark J. Millan and Philippe Marin

Corresponding author: Philippe Marin, Institut de Génomique Fonctionnelle

Review timeline:

Submission date:	30 March 2012
Editorial Decision:	11 May 2012
Revision received:	16 July 2012
Accepted:	31 July 2012

Transaction Report:

(Note: With the exception of the correction of typographical or spelling errors that could be a source of ambiguity, letters and reports are not edited. The original formatting of letters and referee reports may not be reflected in this compilation.)

1st Editorial Decision 11 May 2012

Thank you for the submission of your research manuscript to EMBO Molecular Medicine. We have now heard back from the three referees whom we asked to evaluate your manuscript. As you will see from the reports below, the referees find the topic of your study of potential interest. However, they raise substantial concerns on your work, which should be convincingly addressed in a major revision of the present manuscript.

As you will see below, in particular, all referees ask for better controls and argumentations of the findings. In addition referee #3 would like to see a better co-IP performed in a more controlled way (point C).

Should you be able to address all the referees' criticisms in full, we would be willing to consider a revised manuscript. Please note that it is EMBO Molecular Medicine policy to allow only a single round of revision and that, as acceptance or rejection of the manuscript will depend on another round of review, your responses should be as complete as possible.

Revised manuscripts should be submitted within three months of a request for revision; they will otherwise be treated as new submissions, except under exceptional circumstances in which a short extension is obtained from the editor. Also, the length of the revised manuscript may not exceed 60,000 characters (including spaces) and, including figures, the paper must ultimately fit onto optimally ten pages of the journal. You may consider including any peripheral data (but not methods in their entirety) in the form of Supplementary information.

I look forward to seeing a revised form of your manuscript as soon as possible.

Yours sincerely,

Editor
EMBO Molecular Medicine

***** Reviewer's comments *****

Referee #1 (Comments on Novelty/Model System):

This is an interesting paper describing the connection between 5ht6 receptor activation with mTOR pathway suggesting potential avenues for development of novel treatments for schizophrenia.The following issues need to be better clarified:1-the word social cognition should be explained better,2-the sentence describing 5ht6 stimulation of Gs-adenylate cyclase in page 3 is not clear,3-need to mention which proteins in mTOR pathway were identified in page 3,4-need to define mTOR2 pathway,5-was the administration of WAY181187 acute or chronic as stated in page 6?6-what is the significance of involvement of medium spiny neurons in striatum?7-are there any postmortem evidence for enhanced mTOR signalling in schizophrenia?8-explain the lack of effect in striatum as stated in page 12 ?

Referee #1 (Other Remarks):

following minor revision the paper maybe suitable for publication.

Referee #2:

This is an interesting paper describing the ability of the 5-HT6 receptor to regulate the brain mTOR signaling pathway. The authors took an unbiased approach to identify the putative interactome of the 5-HT6 receptor by first isolating and sequencing candidate interacting proteins in HEK-293 cells. Interestingly, 6 of the 28 interacting protein sequences obtained were components of the mTOR signaling pathway. Using biochemical brain extracts from mice and rats, they document that the 5-HT6 receptor can interact with the identified and other (Rheb) components of the mTOR pathway in a pharmacologically selective fashion. The most interesting aspect of the paper is that the authors then show that manipulating the mTOR pathway with rapamycin can reverse the 5HT6R agonist-induced deficits in social recognition and discrimination and novel object discrimination. The authors then go on to use two rat models of cognitive deficits, the PCP induced deficits in social discrimination and the housing isolation-induced deficit in novel object discrimination to show that much like 5-HT6R antagonists, blocking mTOR signaling by rapamycin reverses these deficits. The authors also show that biochemical indices (mTOR or downstream targets phosphorylation) of modulation of the mTOR pathway can essentially be visualized in GABAergic neurons of the striatum and cortex but that the pharmacological effects on behaviors are mostly mediated by regulation of the pathway in the cortical area as would be expected for cognitive processes.

Minor Comments:

1) The data presented in Fig S2D showing the lack of effect of the C-terminal 5-HT6R are not very convincing since there are still a significant activation of pSer2448mTOR and pSer240/244S6 in the absence of the 5-HT6R C terminal. The raises an issue that is not discussed or speculated upon in the manuscript as to what might be the mechanism by which these effects are carried out.
2) The concluding statement at the end of the discussion that "Irrespective of the outcome, the present work helps to resolve and inter-links two persistent conundrums: namely, of the molecular substrates mediating the detrimental influence of 5-HT6 receptors on cognition, and of cellular events accounting for cognitive deficits in schizophrenia" is too strong and should be tempered as these observations are in animal models that recapitulate schizophrenia-like symptoms. The phrase would be more prudent with a "may help".
3) The experimental design switches back and forth between mice and rats for the biochemistry and behavioral assessments. However, there are, at least not obvious, mentions of why either of the chosen systems is more appropriate. This may be obvious to familiar readers but may not to the general readership of the journal.

Referee #3:

Meffre et al. used immunoprecipitation of HA-5-HT6R and subsequent SDS-PAGE and mass spectrometry to identify interacting partners from HEK 293 cell extracts. Several candidate proteins were identified. The list of identified proteins is striking in that several kinases, particularly of the PI3K superfamily, were found. The authors also found additional mTOR signaling pathway components among their identified proteins. At first glance this is interesting as such proteins are not commonly identified in MS-based protein-protein interaction analyses. The authors then use a series of antibodies and inhibitors toward validation of the interaction of mTOR with 5-HT6R and toward characterizing mTOR signaling before and after stimulation of agonists of 5-HT6R. The authors also examine the effects of 5-HT6R agonists and mTOR pathway inhibitors in vivo, making measurements at cellular and behavioral levels in mice. The authors argue that given their model systems these effects have therapeutic implications for schizophrenia.

Overall the paper is quite interesting and spans basic mechanism to functional/behavioral aspects. While it is an early to claim the importance of their findings toward the treatment of schizophrenia, the results of the study will be interesting to the field. Furthermore, it is of general importance to identify and characterize interacting partners of 5-HT6R. However, several points are listed below that are important to address before publication should be considered:

A. Supplementary Table S1-While the MS analysis appears to be sound, additional information on how this list was generated is needed-the current legend and methods section are insufficient. For example, in the methods the authors indicated that only proteins that were found by two peptides or more in each of four replicates (and not in the control IP) were counted as identified. What then does the Table S1 then represent?...the sum of all the peptides identified in all four replicates and thus the percentage is also cumulative? Also, do the peptides identified mean unique peptides or total? Is the percent coverage my mass or by amino acid number? Furthermore, there are several arrows (indicating distinct bands in Fig. 1A) several more than the number of proteins identified by three peptides or more, this is surprising. Have some proteins been left off the list? Most commonly one sees relatively few proteins identified bywith a high number of peptides and then a tail of many more proteins identified by fewer peptides. This is generally true even if subtracting out background. Also, the title of the table is misleading as it gives the impression that all data (MS and MS/MS) were collected in an FT-ICR cell. Again, clarity in the title and more text in the legend will better serve the reader.

B. While the IP in the presence of the HA peptide is appreciated, it is possible that non-specific background proteins remain in the anti-HA IP lane. Arguably the more appropriate comparison is one that compares anti-HA IPs (without HA peptide) from untransfected versus HA-5HT6R transfected cells. For example, assume a sticky protein, irrelevant to 5HT6R, has some affinity for the anti-HA antibody and that the addition of the HA peptide prevents it from coming down in the IP, should that protein be considered a specific binding partner to 5HT6R just because it was identified in the no-HA peptide lane? Such limitations should be discussed.

C. As it stands now the differences in co-immunoprecipitated mTOR and G-alpha-s between HA-5HT6R, HA-5HT7R and HA-5HT6R (plus or minus 5-HT) are not very convincing. They should be co-immunoprecipitated in the same experiment and run on the same gel side-by-side with exposures the same and without cutting up the lanes and separating them. As a control they should have untransfected cells. It would also be important to blot for another of the identified mTOR pathway proteins (such as RAPTOR)in this same series of side-by-side IPs.

D. Indicate the meaning of "significantly-enriched" in the legend to Fig. 1C.

E. While several phosphospecific antibodies are used to query the mTOR signaling pathway, the authors frequently fail to show the appropriate control blots. For example, while Fig. 2A provides the levels of mTOR (with the levels of mTOR seemingly less at longer 5-HT stimulations?), S6, 4EBP1 and Akt, such controls are not shown for figures 2B, 2C, 2D and S2D. In it not sufficient to imply that because they were shown in Fig. 2A they need not be shown in the others. Clearly the authors quantified some of these controls to get data such as the histograms in Fig. 2B.

F. In the discussion on page 10 the authors use spectral counts to argue that mTOR is more proximal

to the immunoprecipitated HA-5-HT6R than a protein where fewer spectral counts are observed, such as for Raptor. While semi-quantitative, spectral counts need to be taken with caution, particularly as regards the "architecture of the complex." Does mTOR have just as many suitably ionizable, tryptic peptides per length as Raptor? Also, why do the authors draw direct lines to the receptor from both mTOR and Raptor if Raptor binding is considered to be indirect? Furthermore, several proteins were covered in higher percentage than mTOR, are these proteins more proximal to 5HT6R or more relevant?

G. How do the stimulated phosphospecific changes in mTOR pathway members by 5-HT compare to other stimuli such as insulin or EGF? The blots suggest 5-HT and WAY1 give a relatively weak stimulation. Having this comparison will be helpful to those in the signaling field.

H. The authors should include a discussion of how a reader might best interpret and compare the concentrations of drugs and agonists delivered to cultured cells versus what was delivered in vivo. Are they in any way comparable?

1st Revision - Authors' Response 16 July 2012

Referee #1:

1- *The word social cognition should be explained better*

Social cognition refers to a complex set of processes used to acquire, interpret and store information about a subject's social environment, including the identity, intentions and behaviour of others (this is now mentioned in the revised manuscript on page 8, lines 4-6). It can be monitored by a battery of behavioral tasks in rodents. These include the social recognition and the social discrimination procedures used in our study, which incorporate several cognitive components such as attention, short-term and working memory.

2- *The sentence describing 5ht6 stimulation of Gs-adenylate cyclase in page 3 is not clear*

The sentence has been reformulated and split into two sentences for better clarity (see page 3, paragraph 2, lines 10-13)

3- *Need to mention which proteins in mTOR pathway were identified in page 3*

We now mention the proteins of the mTOR pathway identified by mass spectrometry (mTOR, Raptor, Neurofibromin 1 and Vps34) at the end of the introduction (see page 3, 2 last lines)

4- *Need to define mTOR2 pathway*

We have defined the mTORC2 complex and specified that the 5-HT_6 receptor does not interact with proteins specific of this complex (see "Results", page 4, last line, and page 5, lines 1-4).

5- *Was the administration of WAY181187 acute or chronic as stated in page 6?*

WAY181187 was administered 30 min before the animals were sacrificed. This information was indicated in the legend to Fig 3 in the previous version (see legend to Fig 4A page 28 of the revised manuscript).

6- *What is the significance of involvement of medium spiny neurons in striatum?*

The significance of 5-HT_6 receptor-elicited mTOR signaling in medium-sized spiny neurons of the striatum is presently unknown. It was recently discovered that mTOR activation in striatum via Rhes (a striatal-specific small G protein) mediates L-DOPA-induced dyskinesia (Santini et al., Science Signaling, 2:ra36, 2009; Subramaniam et al., Nature Neuroscience, 15:191-193, 2011). It has also been proposed that blockade of mTOR signaling in Huntington disease (HD) due to the sequestration of Rhes by mutant Huntingtin, and the attendant loss of protein translation stimulation, might underlie the pronounced atrophy of the striatum in HD (Subramaniam & Snyder, Neuropharmacology 60: 1187-1192, 2011). Whether 5-HT_6 receptor-operated mTOR signaling in striatum regulates these processes remains to be established. This point is now discussed on page 11 (4 last lines) and 12.

7- *Are there any postmortem evidence for enhanced mTOR signalling in schizophrenia?*

To our knowledge, there is no post-mortem evidence for enhanced mTOR signaling in prefrontal cortex of patients with schizophrenia.

8- *Explain the lack of effect in striatum as stated in page 12*

Several lines of evidence indicate that PCP-induced cognitive deficits as well as those seen in schizophrenia are caused by a dysfunction of a subclass of GABAergic inhibitory interneurons in the prefrontal cortex (parvalbumin-positive interneurons), which may alter the rhythmic activity of projection neurons. This dysfunction might be related to an alteration of gene expression in this particular class of neurons and be responsible for the excessive, 5-HT_6 receptor-operated, mTOR signaling in PFC of rats treated with PCP or housed in isolation. To our knowledge, a comparable dysfunction of GABAergic transmission has not been reported in striatum. We have modified the discussion to address the referee's question (page 13, paragraph 1).

Referee #2

1- The data presented in Fig S2D showing the lack of effect of the C-terminal 5-HT6R are not very convincing since there are still a significant activation of pSer2448mTOR and pSer240/244S6 in the absence of the 5-HT6R C terminal. The raises an issue that is not discussed or speculated upon in the manuscript as to what might be the mechanism by which these effects are carried out.

We now provide a quantification of the data illustrated on Fig. S2D, which indicates that the deletion of the 49 C-terminal residues of the 5-HT_6 receptor strongly affects receptor-elicited mTOR signaling, even though, as noted by the referee, a residual activation of mTOR was detected in cells expressing the truncated receptor (see Fig S2E). This result is consistent with the critical (but not exclusive) role of 5-HT_6 receptor-mTOR physical interaction in the activation of this pathway by the receptor, as it is discussed on page 11 (paragraph 2). The residual mTOR activation by the truncated receptor might reflect the ability of the receptor to engage the PI3K/Akt/Rheb pathway or an alternative signaling pathway upstream to mTOR.

2- The concluding statement at the end of the discussion that "Irrespective of the outcome, the present work helps to resolve and inter-links two persistent conundrums: namely, of the molecular substrates mediating the detrimental influence of 5-HT6 receptors on cognition, and of cellular events accounting for cognitive deficits in schizophrenia" is too strong and should be tempered as these observations are in animal models that recapitulate schizophrenia-like symptoms. The phrase would be more prudent with a "may help".

We agree with the referee's comment and the sentence has been modified (see Discussion, page 13, last sentence).

3- The experimental design switches back and forth between mice and rats for the biochemistry and behavioral assessments. However, there are, at least not obvious, mentions of why either of the chosen systems is more appropriate. This may be obvious to familiar readers but may not to the general readership of the journal.

5-HT_6 receptor-elicited mTOR signaling was systematically analyzed in mice. As noticed by the referee, behavioural studies were carried out in rats, since they were articulated around two core sets of experiments employing complementary neurodevelopmental models of schizophrenia, which are well validated in the rat but not as yet in the mouse. Accordingly, mTOR signaling was analyzed in parallel employing these procedures. These experiments confirmed the ability of 5-HT_6 receptor to activate the mTOR pathway in both rats *and* mice.

Referee #3

A- *Supplementary Table S1-While the MS analysis appears to be sound, additional information on how this list was generated is needed-the current legend and methods section are insufficient. For example, in the methods the authors indicated that only proteins that were found by two peptides or more in each of four replicates (and not in the control IP) were counted as identified. What then does the Table S1 then represent?...the sum of all the peptides identified in all four replicates and thus the percentage is also cumulative? Also, do the peptides identified mean unique peptides or total? Is the percent coverage my mass or by amino acid number? Furthermore,*

there are several arrows (indicating distinct bands in Fig. 1A) several more than the number of proteins identified by three peptides or more, this is surprising. Have some proteins been left off the list? Most commonly one sees relatively few proteins identified by/with a high number of peptides and then a tail of many more proteins identified by fewer peptides. This is generally true even if subtracting out background. Also, the title of the table is misleading as it gives the impression that all data (MS and MS/MS) were collected in an FT-ICR cell. Again, clarity in the title and more text in the legend will better serve the reader.

As stated by the referee, proteins listed in Table S1 are those identified by two peptides or more in each of the four replicates and not detected in control immunoprecipitations. For each protein, the "Peptides" column represents the number of unique peptides identified in one replicate experiment (the one yielding the maximal number of peptides identified). The "Coverage" column represents the sequence covered by the identified peptides (in % of amino acids) in the same replicate. As requested by the referee, we have rewritten the Table legend with more text (see page 16 of Supporting Information). We have also clarified the title according to his comment and specified in the "Materials and Methods" section (page 15 of the manuscript) that fragment ions were detected in the linear trap, whereas precursor masses were determined in the Orbitrap analyzer.

As pointed out by the referee, more protein bands than the number of proteins listed in Table S1 were detected in the immunoprecipitation experiment illustrated on Fig 1A. There are several possible explanations for this: 1) The gel illustrated on this figure corresponds to one experiment and some of the arrows may represent proteins not detected in the four replicate experiments, whereas the list only contains proteins identified in each of the four replicates; 2) several arrows may correspond to the bait (5-HT_6 receptor monomer and dimer). The position of bands where the receptor was the first ranked identified protein (and corresponding to the expected size of receptor monomer and dimer) has been added on the figure.

B- While the IP in the presence of the HA peptide is appreciated, it is possible that non-specific background proteins remain in the anti-HA IP lane. Arguably the more appropriate comparison is one that compares anti-HA IPs (without HA peptide) from untransfected versus HA-5HT6R transfected cells. For example, assume a sticky protein, irrelevant to 5HT6R, has some affinity for the anti-HA antibody and that the addition of the HA peptide prevents it from coming down in the IP, should that protein be considered a specific binding partner to 5HT6R just because it was identified in the no-HA peptide lane? Such limitations should be discussed.

We cannot indeed rule out that some of the identified proteins are irrelevant to 5-HT_6 receptors and that their presence reflects some affinity for the anti-HA antibody. This point is not now mentioned in the "Results" section (page 4, lines 17-20). Moreover, we have performed new co-IP experiments to address Point C by using cells transfected with empty vector in control experiments (see below).

C- As it stands now the differences in co-immunoprecipitated mTOR and G-alpha-s between HA-5HT6R, HA-5HT7R and HA-5HT6R (plus or minus 5-HT) are not very convincing. They should be co-immunoprecipitated in the same experiment and run on the same gel side-by-side with exposures the same and without cutting up the lanes and separating them. As a control they should have untransfected cells. It would also be important to blot for another of the identified mTOR pathway proteins (such as RAPTOR) in this same series of side-by-side IPs.

As requested by the referee, we have performed new co-IP experiments, which were run side-by-side on the same gel (see Fig 1D). As stated above, these experiments were carried out using cells transfected with an empty vector as control. They also show that Raptor and, though not requested by the referee, Neurofibromin 1, another protein located upstream to mTORC1 in the mTOR pathway, specifically co-immunoprecipitate with 5-HT_6 receptors and not with 5-HT_7 receptors. These new data are described in "Results", page 5, lines 9-16).

D- Indicate the meaning of "significantly-enriched" in the legend to Fig. 1C.

We have modified the legend of Fig 1C according to the referee's comment (see page 27).

E- While several phosphospecific antibodies are used to query the mTOR signaling pathway, the authors frequently fail to show the appropriate control blots. For example, while Fig. 2A provides the levels of mTOR (with the levels of mTOR seemingly less at longer 5-HT stimulations?), S6, 4EBP1 and Akt, such controls are not shown for figures 2B, 2C, 2D and S2D. In it not sufficient to imply that because they were shown in Fig. 2A they need not be shown in the others. Clearly the authors quantified some of these controls to get data such as the histograms in Fig. 2B.

We now show control blots performed with antibodies recognizing proteins of interest independently of their phosphorylation state and corresponding to each of the illustrated Western blotting experiments. Note that we split Fig 2 into two figures (Fig 2 and 3, respectively) to keep the images visible (see also Fig S2D and S3) and that none of the treatments used in these experiments significantly affected the total level of studied proteins.

F- In the discussion on page 10 the authors use spectral counts to argue that mTOR is more proximal to the immunoprecipitated HA-5-HT6R than a protein where fewer spectral counts are observed, such as for Raptor. While semi-quantitative, spectral counts need to be taken with caution, particularly as regards the "architecture of the complex." Does mTOR have just as many suitably ionizable, tryptic peptides per length as Raptor?

We agree with the referee that spectral counting should be taken with caution and we only hypothesized in the discussion that Raptor might be recruited indirectly (via mTOR) by 5-HT_6 receptors. As indicated in the previous version of the manuscript, we identified in the four replicates many more peptides in the mTOR sequence (42 unique peptides corresponding to 20.2% sequence coverage) than in the Raptor sequence (5 unique peptides corresponding to 4.7% sequence coverage). It is unlikely that these different values only reflect a difference in the number of suitably ionizable tryptic peptides in both proteins. This is now mentioned in the discussion to take into account the referee's comment (see page 10, 3 lines from bottom).

Also, why do the authors draw direct lines to the receptor from both mTOR and Raptor if Raptor binding is considered to be indirect?

As indicated in the figure legend, dotted lines represent signaling pathways, whereas full lines represent established interactions. We did not discriminate direct and indirect interactions due to the absence of precise information regarding the architecture of the complex.

Furthermore, several proteins were covered in higher percentage than mTOR, are these proteins more proximal to 5HT6R or more relevant?

As noticed by the referee, some proteins were covered in higher percentage than mTOR. These include, for instance, proteins known to be involved in the trafficking of GPCRs, such as clathrin. These proteins are probably relevant to 5-HT_6 receptor function. However, they are not specific of this receptor and have been identified in similar screens performed with other 5-HT receptors (unpublished results). In contrast, as stated by the referee, proteins of the mTOR pathway are not commonly identified by MS-based protein-protein interaction studies, which, together with the possible role of this kinase in cognition, encouraged us to analyze in depth the functional consequence of its recruitment by the receptor.

G- How do the stimulated phosphospecific changes in mTOR pathway members by 5-HT compare to other stimuli such as insulin or EGF? The blots suggest 5-HT and WAY1 give a relatively weak stimulation. Having this comparison will be helpful to those in the signaling field.

We have now compared mTOR stimulation induced by a 5-HT_6 agonist (WAY181187) with those elicited by IGF-1and EGF. As shown in Fig S3, these treatments induced comparable activation of the mTOR pathway in HEK-293 cells. These data, described in "Results" (page 6, lines 4-6), further support the potential physiological relevance of 5-HT_6 receptor-elicited mTOR signaling.

H- The authors should include a discussion of how a reader might best interpret and compare the concentrations of drugs and agonists delivered to cultured cells versus what was delivered in vivo. Are they in any way comparable?

It is difficult to compare the concentrations of drugs delivered to cultured cells with those resulting from their administration *in vivo*. The latter are dependent on drug bioavailability and half-life. All ligands were applied at maximally effective concentrations to cultured cells, based on published concentration-response curves. Likewise, they were administered to animals at doses known to induce robust behavioral responses.
