# Supplementary material for: 5-HT6 receptor recruitment of mTOR as a mechanism for perturbed cognition in schizophrenia
Source: EMBO Mol Med. 2012 Oct 2;4(10):1043–56. doi: 10.1002/emmm.201201410 (PMC3491835; doi:10.1002/emmm.201201410)
Supplement: Supplementary file 2 [file emmm0004-1043-SD2.pdf]

5-HT_6 Receptor Recruitment of mTOR as a Mechanism for Perturbed Cognition in Schizophrenia

Supporting Information

Table of content

Figure S1. Functionality of HA-5-HT_6 receptors expressed in HEK-293 cells 2

Figure S2. 5-HT_6 receptor-elicited mTOR signaling depends on its physical interaction with the C-terminal extremity of the receptor. 3

Figure S3. Comparison of mTOR activation by WAY181187 and growth factors in HEK-293 cells 5

Figure S4. Stimulation of mTOR signaling upon activation of 5-HT_6 receptors in prefrontal cortex and striatum. 6

Figure S5. Accumulation of rapamycin in blood and brain of rats following intraperitoneal administration. 8

Figure S6. Phosphorylation of S6 in DARPP32-negative prefrontocortical neurons and striatal medium-sized spiny neurons of rats treated with WAY181187. 9

Figure S7. Phosphorylation of S6 in GABAergic and non-GABAergic prefrontocortical neurons of rats treated with WAY181187 10

Figure S8. Reversal by SB258585 of the deficits in social novelty discrimination and novel object discrimination induced by WAY181187. 11

Figure S9. Effect of rapamycin upon the deficit in social recognition induced by scopolamine. 13

Figure S10. Lack of effects of WAY181187 and rapamycin on total object exploration in the novel object discrimination procedure. 14

Table S1. Proteins co-immunoprecipitated with the 5-HT_6 receptor and identified by tandem mass spectrometry. 16

Table S2. Results of MS/MS analyses. 17

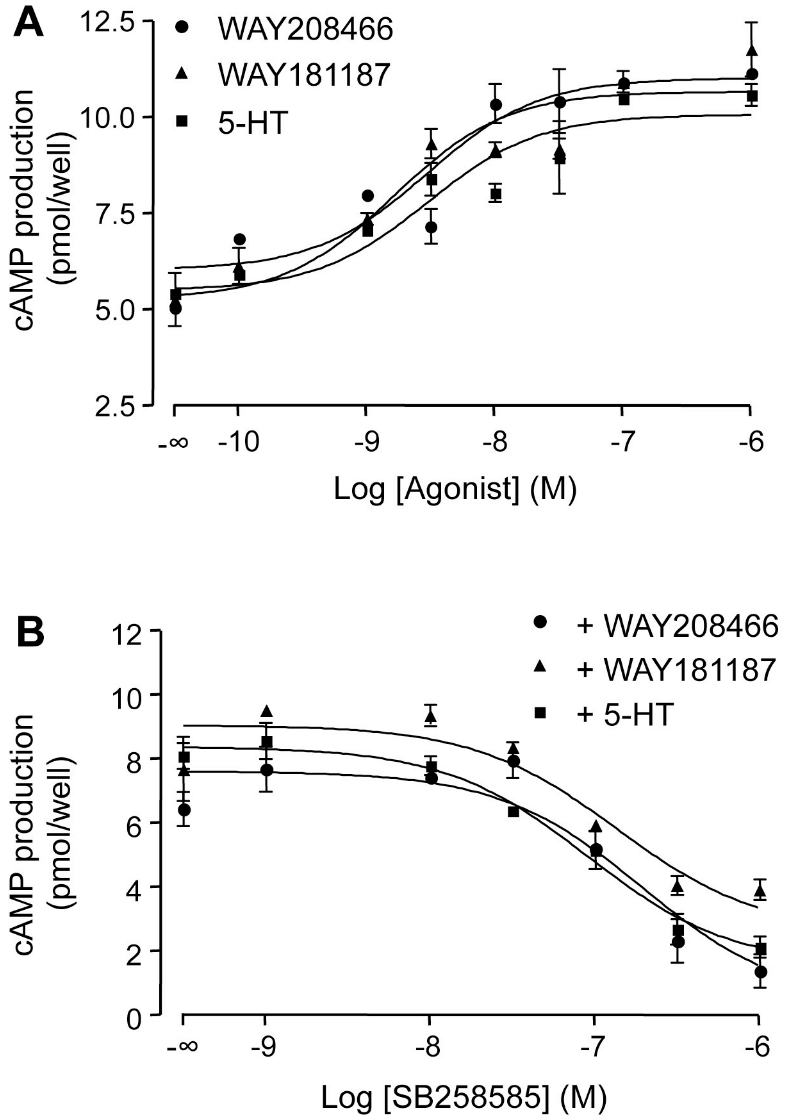

Figure S1. Functionality of HA-5-HT_6 receptors expressed in HEK-293 cells.

HEK-293 cells transiently transfected with the plasmid encoding HA-tagged 5-HT_6 receptor were challenged for 5 min with the indicated concentrations of either 5-HT or WAY181187 or WAY208466 (**A**) or with 30 nM of the corresponding agonists in the presence of increasing concentrations of SB258585 (added 10 min before the agonists, **B**). Treatments were performed in the presence of 0.5 mM of the phosphodiesterase inhibitor 3-isobutyl-1-methyl xantine. cAMP production was quantified by HTRF® using the cAMP Dynamic kit (Cisbio International). Results are the means ± SEM of values obtained in a representative experiment performed in triplicate. Two other experiments performed on different sets of cultured cells yielded similar results.

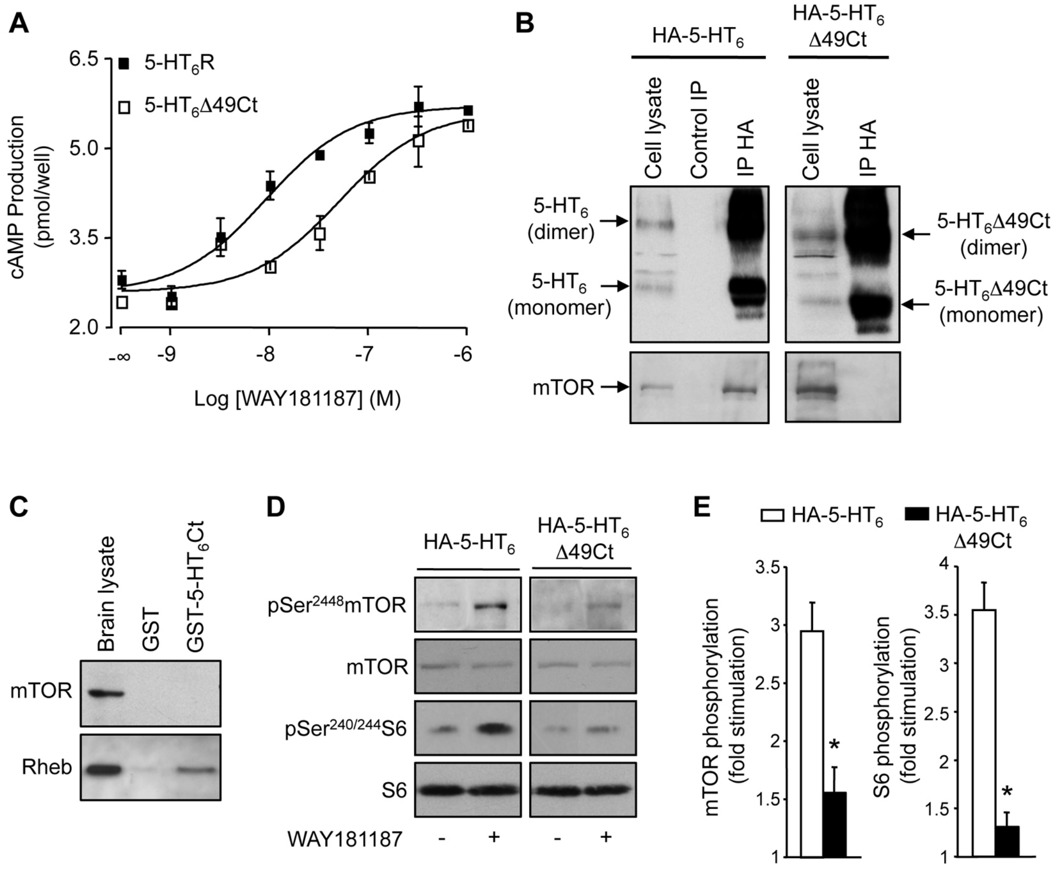

Figure S2. 5-HT$_6$ receptor-elicited mTOR signaling depends on its physical interaction with the C-terminal extremity of the receptor.

A. HEK-293 cells transiently expressing wild type 5-HT$_6$ receptor or a truncated receptor deleted of the 49 C-terminal amino acids (5-HT$_6$Δ49Ct) were exposed to incremental concentrations of WAY181187 in the presence of 0.5 mM 3-isobutyl-1-methylxantine and cAMP production was quantified by HTRF®. Data are means ± SEM of values obtained in a typical experiment performed in triplicate and are representative of three experiments performed on different sets of cultured cells.

B. Analysis of mTOR co-immunoprecipitation with HA-5HT$_6$ and HA-5-HT$_6$Δ49Ct receptors expressed in HEK-293 cells showed that the deletion of the 49 C-terminal residues of the receptor abolished its association with mTOR.

C. GST pull-downs showing the recruitment of Rheb from mice brain but not of mTOR by the 5-HT$_6$ receptor C-terminus. In (**B**) and (**C**), inputs represent 1% of the material used for immunoprecipitations or pull-downs.

D. Cells expressing wild type 5-HT_6 receptor or 5-$HT_6\Delta$49Ct receptor were exposed for 2 min to vehicle or 1 μM WAY181187. WAY181187-elicited mTOR signaling was assessed by phosphorylation of mTOR (Ser^{2448}) and S6 ($Ser^{240/244}$). The data illustrated in (**B-D**) are representative of three independent experiments.

E. Immunoreactive signals were quantified by densitometry. Data represent the stimulatory effect of WAY181187 in cells expressing wild type 5-HT_6 receptor or 5-$HT_6\Delta$49Ct receptor and are the mean ± SEM of values obtained in three independent experiments. * $p<0.05$ *vs*. WAY181187-induced response in cells expressing wild type receptors.

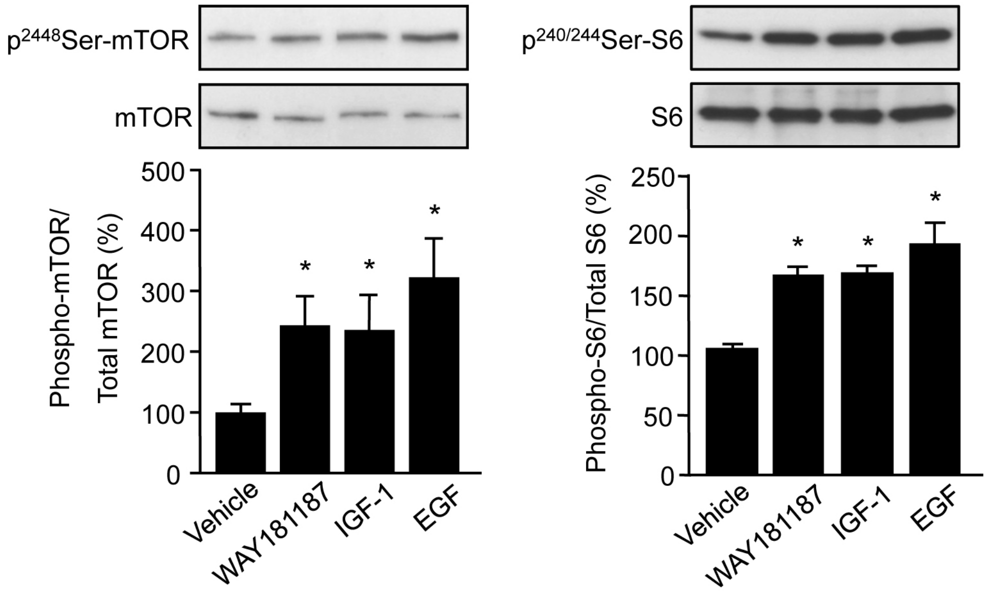

Figure S3. Comparison of mTOR activation by WAY181187 and growth factors in HEK-293 cells.

HEK-293 cells expressing 5-HT_6 receptors were exposed for 2 min to vehicle or WAY181187 (1 μM) or insulin-like growth factor 1 (IGF-1, 500 ng/ml) or epidermal growth factor (EGF, 20 ng/ml). mTOR signaling was assessed by sequential immunoblotting with antibodies against phosphorylated mTOR (Ser^{2448}) and S6 ($Ser^{240/244}$) and with antibodies recognizing these proteins independently of their phosphorylation state. The illustrated blots are representative of three independent experiments performed on different cultures. Immunoreactive signals were quantified by densitometry. Data, expressed as ratios of phosphorylated to total proteins, represent the means ± SEM of values obtained in three independent experiments. * $p<0.05$ *vs*. vehicle.

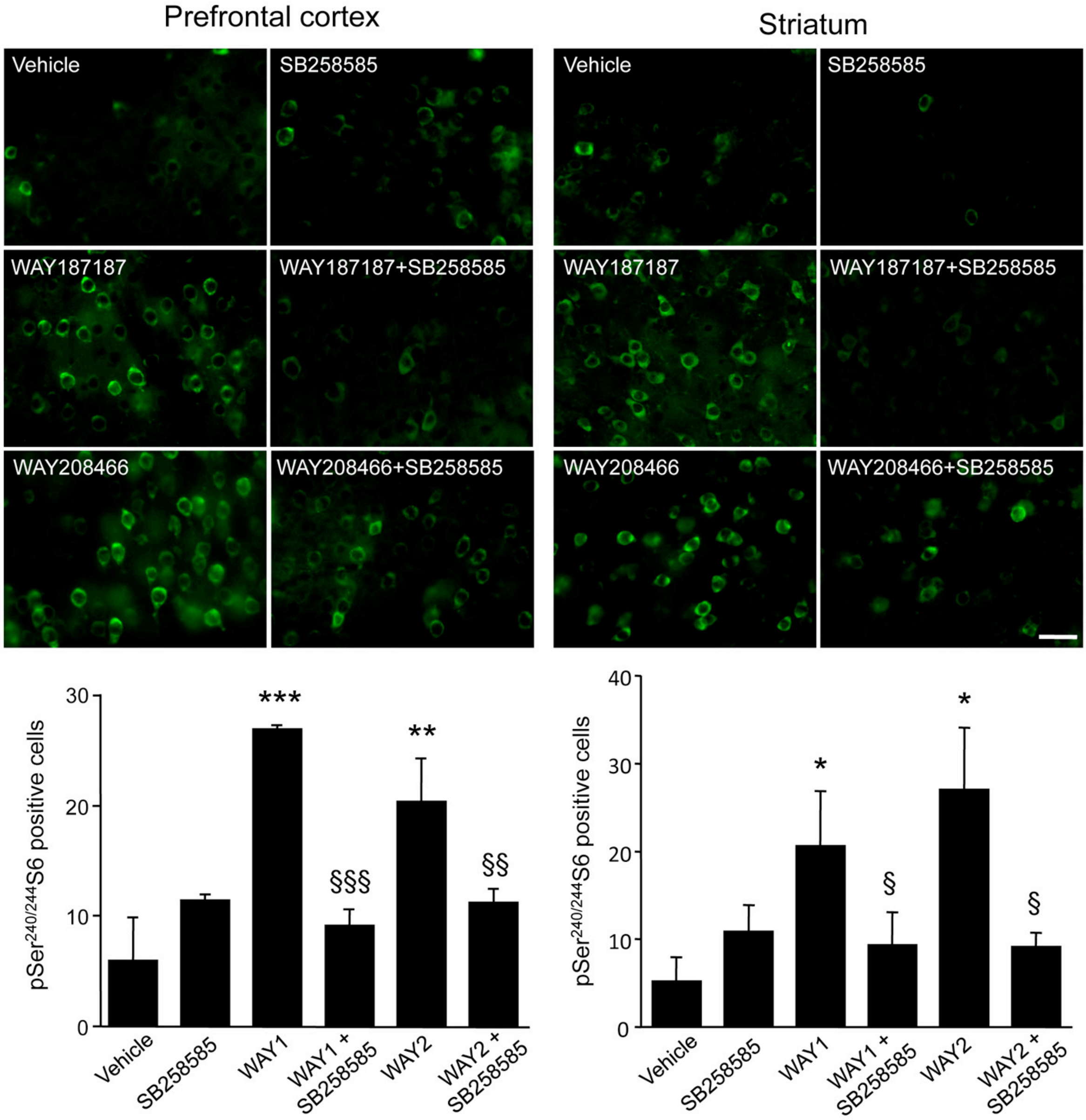

Figure S4. Stimulation of mTOR signaling upon activation of 5-HT$_6$ receptors in prefrontal cortex and striatum.

Immunofluorescent detection of cells positive for phospho-Ser$^{240/244}$-S6 in the prefrontal cortex and striatum of mice treated for 30 min with either Vehicle or WAY181187 (WAY1, 10 mg/kg, i.p.) or WAY208466 (WAY2, 10 mg/kg, i.p.) or SB258585 (10 mg/kg, i.p., alone or in combination with WAY181187 or WAY208466). SB258585 was injected 30 min before agonists. Scale bar: 40 µm. Quantification was performed on 448 µm x 335 µm images. Values represent the means ± SEM of counting performed in n = 4-6 mice. Statistical

significance was determined by ANOVA followed by Newman-Keuls test. * $p<0.05$, *** $p<0.01$, *** $p<0.001$ *vs*. vehicle-treated rats, § $p<0.05$, §§ $p<0.01$, §§§ $p<0.001$ *vs*. WAY181187 or WAY208466 alone.

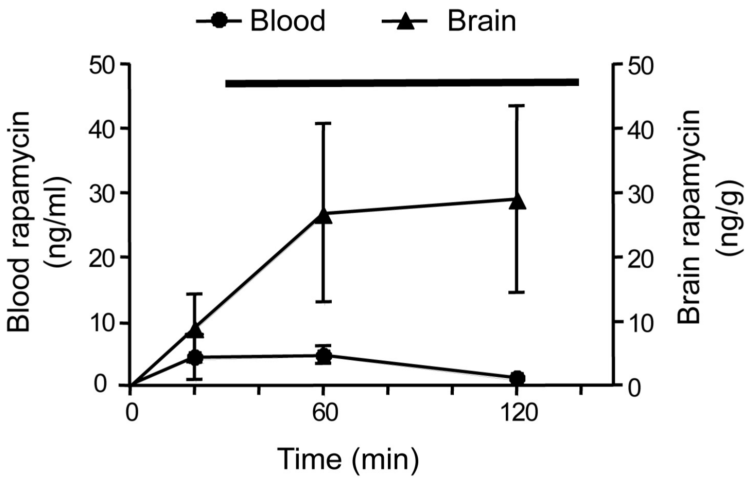

Figure S5. Accumulation of rapamycin in blood and brain of rats following intraperitoneal administration.

Rats that received intraperitoneal injection of rapamycin (10 mg/kg) were sacrificed by decapitation 20, 60 or 120 min after the injection. The blood was collected in chilled tubes containing heparin lithium as an anticoagulant. The plasma was separated by centrifugation (1,000 x g, 20 min, 4°C) and frozen at -80°C. The brains were quickly removed, frozen on a slab of dry ice and stored at -80°C until determination of rapamycin content. Rapamycin was quantified by LC-MS. The black bar indicates the time frame during which behavioral studies were performed. Data are means ± SEM of values obtained in three rats.

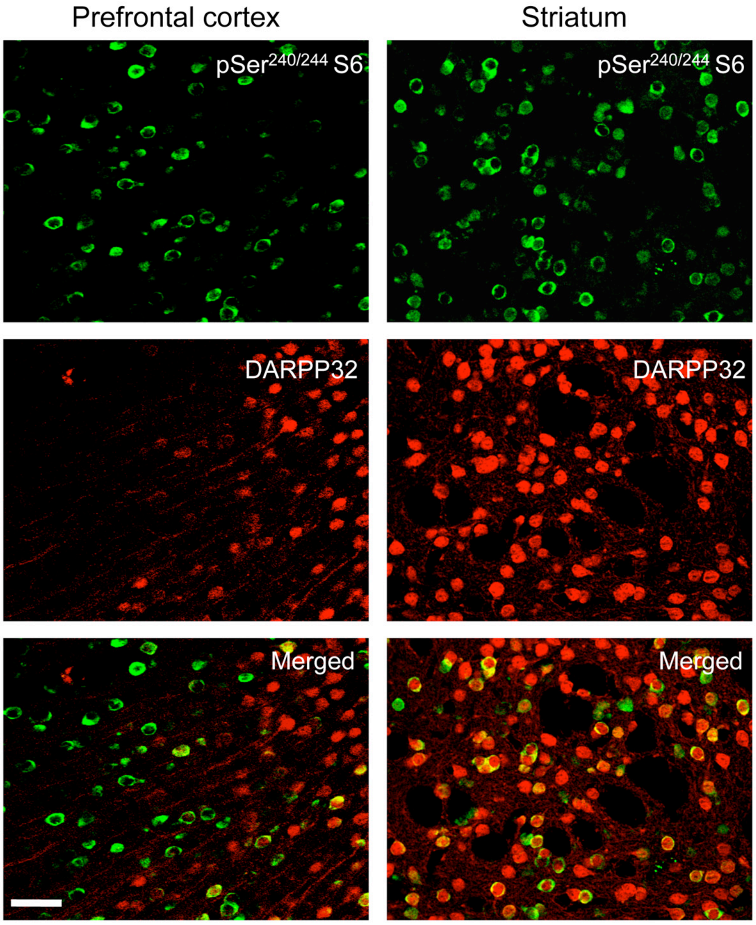

Figure S6. Phosphorylation of S6 in DARPP32-negative prefrontocortical neurons and striatal medium-sized spiny neurons of rats treated with WAY181187.

Immunofluorescent staining of phospho-Ser$^{240/244}$ S6 (green) and DARPP32 (red) in prefrontal cortex (left) and striatum (right) of rats treated with WAY181187 (10 mg/kg i.p., 30 min) was visualized by confocal microscopy. Scale bar: 40 μm. More than 90% of phospho-S6-stained cells in striatum of rats treated with WAY181187 were also positive for DARPP32 (4 fields originating from different animals counted), whereas the majority of phospho-S6 positive cells in prefrontal cortex were DARPP32-negative.

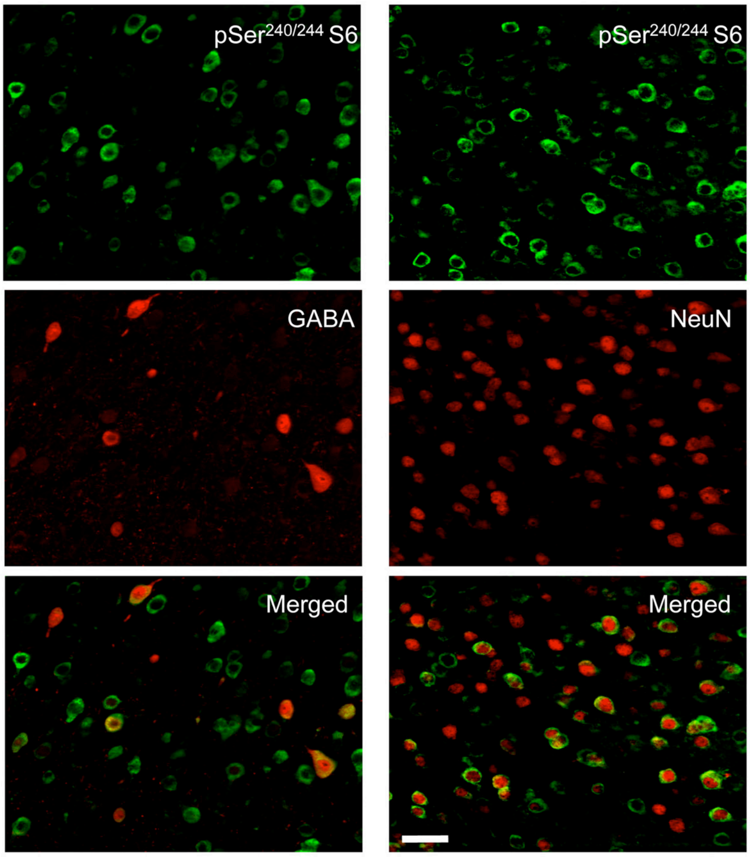

Figure S7. Phosphorylation of S6 in GABAergic and non-GABAergic prefrontocortical neurons of rats treated with WAY181187.

Immunofluorescent staining of phospho-Ser$^{240/244}$ S6 (green) and NeuN (red) or GABA (red) in prefrontal cortex of rats treated with WAY181187 (10 mg/kg i.p., 30 min) was visualized by confocal microscopy. Scale bar: 40 µm. Note that phospho-Ser$^{240/244}$S6 immunostaining was detected in both GABAergic and non-GABAergic neurons.

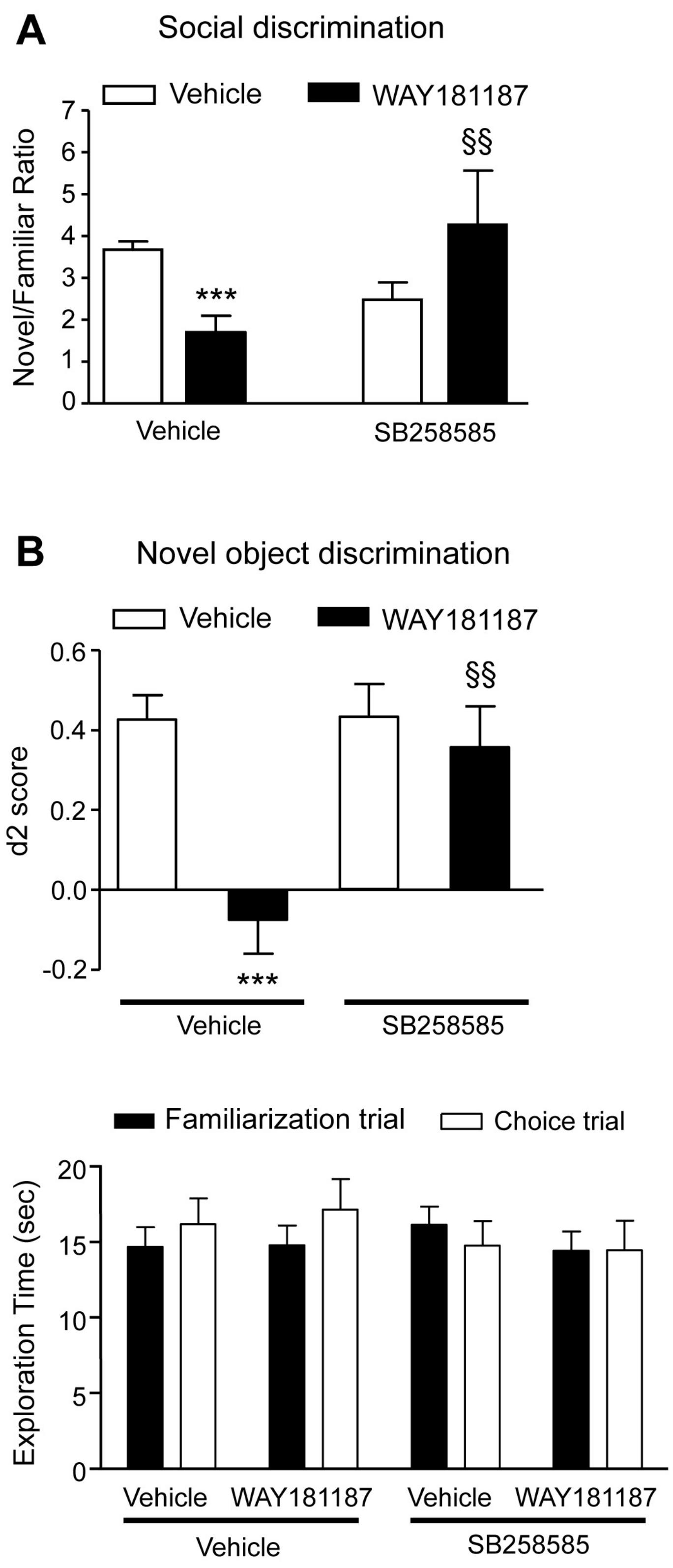

Figure S8. Reversal by SB258585 of the deficits in social novelty discrimination and novel object discrimination induced by WAY181187.

A. Rats injected with vehicle or SB258585 (10 mg/kg i.p., injected 45 min before the first session of the test) were then treated with vehicle or WAY181187 (10 mg/kg i.p.

injected 30 min before the first session). Data, expressed as ratios of time spent investigating the novel juvenile rat to time spent investigating the familiar one during the second 5-min session, are means ± SEMs (n = 7-8 rats per group). *** $p<0.001$ *vs*. vehicle/vehicle, §§ $p<0.01$ *vs*. vehicle/WAY181187.

B. Rats injected with vehicle or SB258585 (10mg/kg s.c., 45 min prior to familiarization trial) were then treated with WAY181187 (2.5 mg/kg s.c., 30 min prior to the familiarization trial). Performance in the novel object discrimination task was assessed by the d2 score (n = 12, upper panel). *** $p<0.001$ *vs*. vehicle/vehicle, §§ $p<0.01$ *vs*. vehicle/WAY181187. Neither SB258585 nor WAY181187, alone or in combination, significantly alter total object exploration in both the familiarization and choice trials ($p>0.05$, bottom panel).

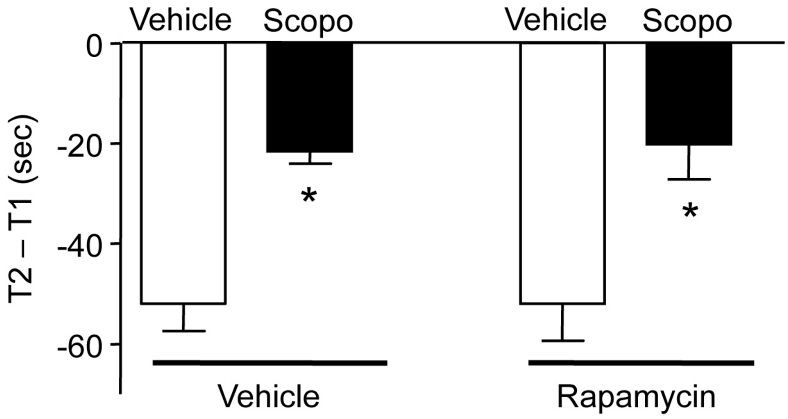

Figure S9. Effect of rapamycin upon the deficit in social recognition induced by scopolamine.

Rats were pre-treated by either vehicle or rapamycin (10 mg/kg i.p., 45 min before the test) and then by either vehicle or scopolamine (Scopo, 1.25 mg/kg i.p., 30 min before the test). Data are means ± SEMs (n = 5-6 rats per group) and represent the difference in duration of social investigation between the two 5-min sessions of the test performed without an inter-session delay (T2-T1). * $p<0.05$ *vs*. corresponding vehicle.

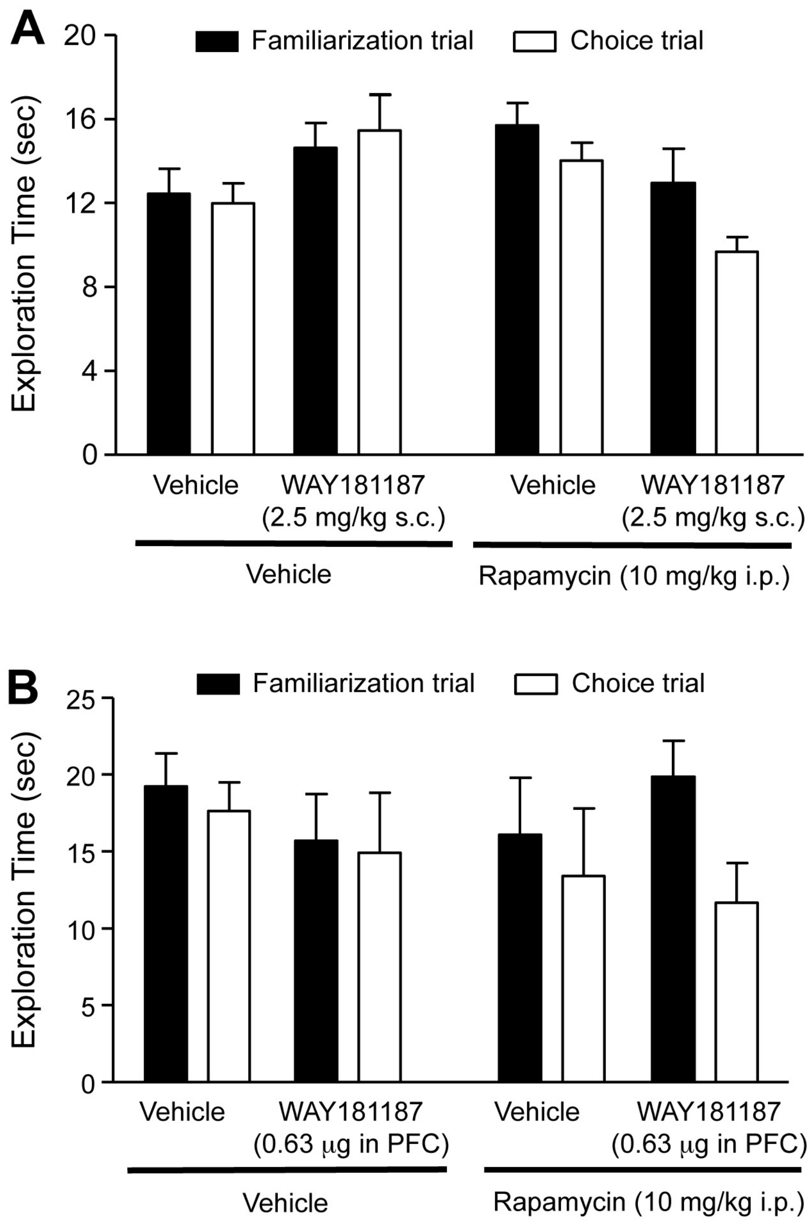

Figure S10. Lack of effect of WAY181187 and rapamycin on total object exploration in the novel object discrimination procedure.

- **A.** Adult rats were injected 45 min prior to the familiarization trial with vehicle or WAY181187 (2.5 mg/kg s.c.) and then with vehicle or rapamycin (10 mg/kg i.p.) 30 min before the familiarization trial. Data are means ± SEMs (n = 12).
- **B.** Rats were implanted with a bilateral cannula above the PFC one week before the test. They were injected with either rapamycin (10mg/kg i.p.) or vehicle 30 min prior to the familiarization trial. Twenty-five min later, WAY181187 (0.63 μg/side) or vehicle (1

μL) was injected bilaterally into the PFC. Data are means ± SEMs (n = 6-7 rats per group). There was no significant main effect of rapamycin or WAY181187 (injected locally or systemically) on total object exploration during either the familiarization trial or the choice trial ($p>0.05$).

Table S1. Proteins co-immunoprecipitated with the 5-HT$_6$ receptor and identified by tandem mass spectrometry.

Protein ID	UniProt Acc	Approved protein name	Peptides	Coverage (%)
5HT6R_HUMAN	P50406	5-hydroxytryptamine receptor 6	11	32,7
ATM_HUMAN	Q13315	Serine-protein kinase ATM	17	6.9
ATR_HUMAN	Q13535	Serine/threonine-protein kinase ATR	3	1.2
ATX10_HUMAN	Q9UBB4	Ataxin-10	14	25.1
C1TM_HUMAN	Q6UB35	Monofunctional C1-tetrahydrofolate synthase	14	17.9
CAND2_HUMAN	O75155	Cullin-associated NEDD8-dissociated protein 2	5	4.5
CDK5_HUMAN	Q00535	Cyclin-dependent kinase 5	4	15.4
CK5P3_HUMAN	Q96JB5	CDK5 regulatory subunit-associated protein 3	6	13.2
CLH1_HUMAN	Q00610	Clathrin heavy chain 1	83	46.1
CND3_HUMAN	Q9BPX3	Condensin complex subunit 3	7	8.7
COG3_HUMAN	Q96JB2	Conserved oligomeric Golgi complex subunit 3	4	4.2
COPG2_HUMAN	Q9UBF2	Coatomer subunit gamma-2	10	13.2
DYN2_HUMAN	P50570	Dynamin-2	7	9.5
EXOC2_HUMAN	Q96KP1	Exocyst complex component 2	10	12.1
IPO8_HUMAN	O15397	Importin-8	8	9.7
TTI1_HUMAN	O43156	TEL2-interacting protein 1 homolog	3	4.3
MTOR_HUMAN	P42345	Serine/threonine-protein kinase mTOR	29	13.8
NCDN_HUMAN	Q9UBB6	Neurochondrin	5	8.2
NF1_HUMAN	P21359	Neurofibromin	20	8.8
NSF_HUMAN	P46359	Vesicle-fusing ATPase	13	19.5
PK3C3_HUMAN	Q8NEB9	Phosphatidylinositol 3-kinase catalytic subunit type 3 (vps34)	3	4.3
RNBP6_HUMAN	O60518	Ran-binding protein 6	6	6.7
RPTOR_HUMAN	Q8N122	Regulatory-associated protein of mTOR	5	4.7
SMG1_HUMAN	Q96Q15	Serine/threonine-protein kinase SMG1	6	2
TBCD_HUMAN	Q9BTW9	Tubulin-specific chaperone D	10	11.2
TELO2_HUMAN	Q9Y4R8	Telomere length regulation protein TEL2 homolog	9	11.8
TNPO1_HUMAN	Q92973	Transportin-1	10	14.7
XPO4_HUMAN	Q9C0E2	Exportin-4	17	15.6
XPOT_HUMAN	O43592	Exportin-T	25	25.6

The list of proteins identified by MS/MS with two peptides or more in each of the four replicate experiments (and not detected in control immunoprecipitations) is depicted. For each protein, the number of unique peptides identified in one replicate experiment (the one yielding the maximal number of peptides identified) and the corresponding sequence coverage (in % of total amino acid number) are indicated.

Table S2. **Results of MS/MS analyses.**

5HT6R: 11 peptides. Peptide coverage: 32.7 %

Peptide	Start	End	Score	M/z (Obs)	Mr (Exp)	Mr (calc)	Miss	Sequence
1	125	133	48.7	544.35	1086.68	1086.68	0	R.YLLILSPLR.Y
2	217	241	32.9	873.12	2616.35	2616.35	1	R.KQAVQVASLTTGMASQASETLQVPR.T + Oxidation (M:13)
3	217	241	19.5	867.79	2600.36	2600.36	1	R.KQAVQVASLTTGMASQASETLQVPR.T
4	218	241	125.8	1237.14	2472.26	2472.26	0	K.QAVQVASLTTGMASQASETLQVPR.T
5	218	241	108.8	1245.14	2488.26	2488.26	0	K.QAVQVASLTTGMASQASETLQVPR.T + Oxidation (M:12)
6	242	253	46.9	424.55	1270.63	1270.63	0	R.TPRPGVESADSR.R
7	343	354	23.2	657.86	1313.70	1313.71	1	R.ERQASLASPSLR.T
8	345	354	46.5	515.29	1028.56	1028.56	0	R.QASLASPSLR.T
9	355	393	57	1281.30	3840.89	3840.89	0	R.TSHSGPRPGLSLQQVLPLPLPPDSDSDSDAGSGGSSGLR.L
10	394	413	56.4	1066.09	2130.17	2130.17	0	R.LTAQLLLPGEATQDPPLPTR.A
11	414	440	53.5	957.83	2870.47	2870.47	0	R.AAAAVNFFNIDPAEPELRPHPLGIPTN.-

ATM : 17 peptides. Peptide coverage: 6.9 %

Peptide	Start	End	Score	M/z (Obs)	Mr (Exp)	Mr (Calc)	Miss	Sequence
1	77	90	44.4	715.39	1428.77	1428.77	0	R.IAKPNVSASTQASR.Q
2	174	184	18.8	452.59	1354.74	1354.74	0	R.LYLKPSQDVHR.V
3	363	375	58.7	757.38	1512.74	1512.74	0	R.SLEISQSYTTTQR.E
4	798	805	30.9	455.76	909.51	909.51	0	K.IASGFFLR.L
5	1634	1646	27	701.84	1401.66	1401.66	0	R.ASQDNPQDGIMVK.L
6	1647	1656	53.2	563.86	1125.71	1125.71	0	K.LVVNLLQLSK.M
7	1974	1992	77.4	1000.99	1999.96	1999.96	0	R.SLAFEEGSQSTTISSLSEK.S
8	2393	2400	33.3	522.74	1043.47	1043.47	0	R.FSDTQYQR.I
9	2444	2453	43.1	600.83	1199.64	1199.64	0	R.ELELDELALR.A
10	2516	2526	52.6	661.87	1321.72	1321.72	0	K.FLPLMYQLAAR.M
11	2590	2604	36.9	573.93	1718.78	1718.78	1	K.QSSQLDEDRTEAANR.I
12	2611	2618	19.6	515.29	1028.57	1028.57	1	R.SRRPQMVR.S
13	2671	2687	18.1	636.65	1906.94	1906.94	0	K.VDHTGEYGNLVTIQSFK.A
14	2839	2848	36	633.33	1264.65	1264.65	0	K.FLDPAIWFEK.R
15	2855	2871	19.1	569.98	1706.92	1706.92	0	R.SVATSSIVGYILGLGDR.H
16	2913	2928	35.6	832.92	1663.83	1663.82	0	R.DIVDGMGITGVEGVFR.R
17	2994	3004	27	640.81	1279.6	1279.6	0	R.NLSDIDQSFNK.V

ATR: 3 peptides. Peptide coverage: 1.2 %

Peptide	Start	End	Score	M/z (Obs)	Mr (Exp)	Mr (Calc)	Miss	Sequence
1	1006	1015	23	478.78	955.54	955.54	0	K.ASPAASALIR.T
2	1291	1300	17.6	590.81	1179.6	1179.6	0	K.AIQHENVDVR.I
3	2575	2587	73.9	700.36	1398.7	1398.7	0	K.APLNETGEVVNEK.A

ATX10: : 14 peptides. Peptide coverage: 25.1 %

Peptide	Start	End	Score	M/z (Obs)	Mr (Exp)	Mr (Calc)	Miss	Sequence
1	11	27	26.5	913.00	1823.98	1823.98	0	R.LSGVMVPAPIQDLEALR.A + Oxidation (M:5)
2	11	27	25.3	905.00	1807.99	1807.99	0	R.LSGVMVPAPIQDLEALR.A
3	120	133	19.4	564.31	1689.91	1689.90	1	R.ELRVEQESLLTAFR.C
4	123	133	68	646.85	1291.68	1291.68	0	R.VEQESLLTAFR.C
5	227	237	38.8	623.83	1245.64	1245.64	0	K.SPELVQAMFPK.L
6	227	237	45.8	631.83	1261.64	1261.64	0	K.SPELVQAMFPK.L + Oxidation (M:8)
7	244	253	50.3	558.84	1115.66	1115.66	0	R.VTLLDLMIAK.I
8	244	253	28.1	566.84	1131.66	1131.66	0	R.VTLLDLMIAK.I + Oxidation (M:7)
9	263	270	23.7	487.77	973.52	973.52	0	K.DDIPVFLR.H
10	289	305	67.3	928.45	1854.89	1854.88	0	K.LASEEPPDDEEALATIR.L
11	359	371	68	661.32	1320.63	1320.63	0	R.AEGDISNVANGFK.S
12	424	438	74.5	851.92	1701.82	1701.82	0	R.NLTEDNSQNQDLIAK.M
13	439	452	39.6	766.90	1531.79	1531.79	1	K.MEEQGLADASLLKK.V
14	439	451	57.9	702.86	1403.70	1403.70	0	K.MEEQGLADASLLK.K

C1TM: 14 peptides. Peptide coverage: 17.9 %

Peptide	Start	End	Score	M/z (Obs)	Mr (Exp)	Mr (Calc)	Miss	Sequence
1	84	92	26	529.81	1057.60	1057.60	0	K.EVLSLLQEK.N
2	178	189	44.2	623.32	1244.63	1244.62	0	K.DVDGVTDINLGK.L
3	206	213	21.6	457.78	913.55	913.55	0	K.AVIELLEK.S
4	353	367	39.7	825.96	1649.90	1649.90	0	K.LQPLSPVPSDIEISR.G
5	380	393	57	781.92	1561.83	1561.82	0	K.EIGLLADEIEIYGK.S
6	414	429	59.6	807.96	1613.90	1613.90	0	K.YVLVAGITPTPLGEGK.S
7	522	530	30.1	483.81	965.60	965.60	0	R.LVPLVNGVR.E
8	531	539	30.9	546.79	1091.56	1091.56	0	R.EFSEIQLAR.L
9	548	560	37.5	718.35	1434.67	1434.67	0	K.TDPSTLTEEEVSK.F
10	564	575	36.1	722.88	1443.74	1443.74	0	R.LDIDPSTITWQR.V
11	639	659	55.9	1037.05	2072.08	2072.08	0	K.SGQPVTADDLGVTGALTVLMK.D
12	749	764	36.9	768.42	1534.83	1534.83	1	K.MHGGGPSVTAGVPLKK.E
13	881	891	29.8	600.81	1199.60	1199.60	0	K.DIELSPEAQAK.I
14	926	935	40	587.83	1173.64	1173.64	0	R.DFILPISDVR.A

CAND2: 5 peptides. Peptide coverage: 4.5 %

Peptide	Start	End	Score	M/z (Obs)	Mr (Exp)	Mr (Calc)	Miss	Sequence
1	227	235	24.7	471.27	940.53	940.53	0	R.VPTSPTAIR.T
2	236	246	56.5	602.33	1202.64	1202.64	0	R.TLIQCLGSVGR.Q
3	864	878	26.6	778.42	1554.83	1554.83	0	K.AVLLEALGSPSEDVR.A
4	1070	1077	17.9	476.73	951.44	951.44	0	R.EVEMGPFK.H + Oxidation (M:4)
5	1184	1196	36.2	641.39	1280.77	1280.77	0	R.AVAALLTIPEVGK.S

CDK5: 4 peptides. Peptide coverage: 15.4 %

Peptide	Start	End	Score	M/z (Obs)	Mr (Exp)	Mr (Calc)	Miss	Sequence
1	218	232	31.6	859.43	1716.84	1716.84	0	R.LLGTPTEEQWPSMTK.L
2	233	254	34.2	831.77	2492.30	2492.30	0	K.LPDYKPYPMYPATTSLVNVVPK.L
3	233	254	19.9	837.11	2508.30	2508.30	0	K.LPDYKPYPMYPATTSLVNVVPK.L + Oxidation (M:9)
4	261	268	20.1	478.79	955.57	955.57	0	R.DLLQNLLK.C

CK5P3: 6 peptides. Peptide coverage: 13.2 %

Peptide	Start	End	Score	M/z (Obs)	Mr (Exp)	Mr (Calc)	Miss	Sequence
1	16	23	47.7	515.29	1028.57	1028.57	0	K.LLDWLVDR.R
2	107	120	62.5	811.44	1620.87	1620.87	0	K.DNTYLVELSSLLVR.N
3	121	130	28.3	588.82	1175.62	1175.62	0	R.NVNYEIPSLK.K

4	337	350	37.8	789.90	1577.79	1577.79	0	R.GPDALTLLEYTETR.N
5	433	440	29.7	496.77	991.53	991.53	0	R.VTEFLQQK.L
6	494	506	32.2	697.86	1393.70	1393.70	0	R.YSGRPVNLMGTSL.-

CLH1: 83 peptides. Peptide coverage: 46.1 %

Peptide	Start	End	Score	M/z (Obs)	Mr (Exp)	Mr (Calc)	Miss	Sequence
1	9	36	48	1054.53	3160.56	3160.55	0	R.FQEHLQLQNLGINPANIGFSTLTMESDK.F + Oxidation (M:24)
2	9	36	66.8	1049.19	3144.56	3144.55	0	R.FQEHLQLQNLGINPANIGFSTLTMESDK.F
3	42	63	38.4	818.09	2451.24	2451.24	1	R.EKVGEQAQVVIIDMNDPSNPIR.R
4	44	63	70.1	1098.06	2194.11	2194.11	0	K.VGEQAQVVIIDMNDPSNPIR.R
5	44	63	102.8	1106.06	2210.10	2210.10	0	K.VGEQAQVVIIDMNDPSNPIR.R + Oxidation (M:12)
6	64	78	40.1	779.41	1556.80	1556.80	0	R.RPISADSAIMNPASK.V
7	64	78	38.4	787.40	1572.79	1572.79	0	R.RPISADSAIMNPASK.V + Oxidation (M:10)
8	87	96	49.5	626.84	1251.65	1251.65	0	K.TLQIFNIEMK.S + Oxidation (M:9)
9	87	96	45.9	618.84	1235.66	1235.66	0	K.TLQIFNIEMK.S
10	101	112	45.3	734.33	1466.65	1466.65	0	K.AHTMTDDVTFWK.W + Oxidation (M:4)
11	101	112	45.1	726.34	1450.66	1450.66	0	K.AHTMTDDVTFWK.W
12	113	140	51.9	1058.86	3173.55	3173.55	0	K.WISLNTVALVTDNAVYHWSMEGESQPVK.M
13	164	176	71.8	750.42	1498.83	1498.83	0	K.WLLLTGISAQQNR.V
14	177	189	27.6	733.40	1464.78	1464.78	1	R.VVGAMQLYSVDRK.V
15	177	188	81.8	669.35	1336.68	1336.68	0	R.VVGAMQLYSVDR.K
16	177	188	59.5	677.35	1352.68	1352.68	0	R.VVGAMQLYSVDR.K + Oxidation (M:5)
17	189	205	82.5	922.99	1843.96	1843.96	1	R.KVSQPIEGHAASFAQFK.M
18	190	205	76.4	858.94	1715.86	1715.86	0	K.VSQPIEGHAASFAQFK.M
19	228	245	53.6	973.03	1944.05	1944.05	0	K.LHIIEVGTPPTGNQPFPK.K
20	246	269	32.8	908.45	2722.34	2722.33	1	K.KAVDVFFPPEAQNDFPVAMQISEK.H + Oxidation (M:19)
21	246	269	64.9	903.12	2706.34	2706.34	1	K.KAVDVFFPPEAQNDFPVAMQISEK.H
22	247	269	76.1	1290.13	2578.24	2578.24	0	K.AVDVFFPPEAQNDFPVAMQISEK.H
23	247	269	30.7	865.75	2594.24	2594.24	0	K.AVDVFFPPEAQNDFPVAMQISEK.H + Oxidation (M:18)
24	270	278	35.2	536.31	1070.61	1070.61	0	K.HDVVFLITK.Y
25	298	320	99.1	1177.13	2352.25	2352.24	0	R.ISGETIFVTAPHEATAGIIGVNR.K
26	355	366	78.9	652.83	1303.65	1303.65	0	R.NNLAGAEELFAR.K
27	367	382	90.3	879.95	1757.88	1757.87	1	R.KFNALFAQGNYSEAAK.V
28	368	382	59.8	815.90	1629.78	1629.78	0	K.FNALFAQGNYSEAAK.V
29	401	429	59.1	1594.35	3186.68	3186.67	0	R.FQSVPAQPGQTSPLLQYFGILLDQGQLNK.Y
30	469	481	62.5	717.40	1432.79	1432.79	0	K.SVDPTLALSVYLR.A
31	507	519	51	804.45	1606.89	1606.89	1	K.KVGYTPDWIFLLR.N
32	508	519	56	740.40	1478.79	1478.79	0	K.VGYTPDWIFLLR.N
33	572	583	34.5	456.90	1367.69	1367.69	0	K.NNRPSEGPLQTR.L
34	584	610	23.2	782.88	3127.51	3127.50	0	R.LLEMNLMHAPQVADAILGNQMFTHYDR.A
35	584	610	29.9	1048.84	3143.50	3143.50	0	R.LLEMNLMHAPQVADAILGNQMFTHYDR.A + Oxidation (M:21)
36	584	610	33.8	1048.84	3143.50	3143.50	0	R.LLEMNLMHAPQVADAILGNQMFTHYDR.A + Oxidation (M:7)
37	584	610	29.6	1054.17	3159.50	3159.49	0	R.LLEMNLMHAPQVADAILGNQMFTHYDR.A + Ox (M:4.7)
38	584	610	29.1	1059.50	3175.49	3175.49	0	R.LLEMNLMHAPQVADAILGNQMFTHYDR.A + Ox (M:4.7.21)
39	626	638	47.3	540.95	1619.83	1619.83	1	R.ALEHFTDLYDIKR.A
40	626	637	36.5	732.87	1463.73	1463.73	0	R.ALEHFTDLYDIK.R
41	669	676	20.1	446.24	890.46	890.46	0	R.AMLSANIR.Q + Oxidation (M:2)
42	669	676	22.3	438.24	874.47	874.47	0	R.AMLSANIR.Q
43	689	706	68.6	1100.05	2198.09	2198.09	0	K.YHEQLSTQSLIELFESFK.S
44	799	806	29.9	528.29	1054.57	1054.57	0	K.YIEIYVQK.V
45	831	837	26.6	413.78	825.54	825.54	0	K.NLILVVR.G
46	838	852	83.5	854.43	1706.85	1706.85	1	R.GQFSTDELVAEVEKR.N
47	838	851	74.7	776.38	1550.75	1550.75	0	R.GQFSTDELVAEVEK.R
48	857	865	33.7	555.84	1109.66	1109.66	0	K.LLLPWLEAR.I
49	882	892	62.6	667.82	1333.63	1333.63	0	K.IYIDSNNNPER.F
50	896	903	19.4	522.23	1042.44	1042.44	0	R.ENPYYDSR.V
51	951	967	77.5	1002.02	2002.02	2002.02	1	R.KDPELWGSVLLESNPYR.R
52	968	993	41.5	961.18	2880.51	2880.51	0	R.RPLIDQVVQTALSETQDPEEVSVTVK.A
53	994	1010	57.9	974.01	1946.01	1946.01	0	K.AFMTADLPNELIELLEK.I
54	994	1010	56.8	655.01	1962.00	1962.00	0	K.AFMTADLPNELIELLEK.I + Oxidation (M:3)
55	1011	1022	79.9	708.37	1414.72	1414.72	0	K.IVLDNSVFSEHR.N
56	1023	1034	67.3	677.43	1352.84	1352.84	0	R.NLQNLLILTAIK.A
57	1040	1046	29.6	462.73	923.45	923.45	0	R.VMEYINR.L
58	1040	1046	28.9	470.73	939.45	939.45	0	R.VMEYINR.L + Oxidation (M:2)
59	1074	1094	58.7	790.09	2367.26	2367.25	1	R.KFDVNTSAVQVLIEHIGNLDR.A
60	1075	1094	32.4	747.39	2239.16	2239.16	0	K.FDVNTSAVQVLIEHIGNLDR.A
61	1123	1130	19.7	469.75	937.48	937.48	0	K.EAIDSYIK.A
62	1131	1155	76.3	1370.62	2739.23	2739.23	0	K.ADDPSSYMEVVQAANTSGNWEELVK.Y
63	1131	1155	83.9	1378.62	2755.23	2755.23	0	K.ADDPSSYMEVVQAANTSGNWEELVK.Y + Oxidation (M:8)
64	1166	1179	76.2	806.93	1611.84	1611.84	0	R.ESYVETELIFALAK.T
65	1183	1204	48.4	822.08	2463.22	2463.21	0	R.LAELEEFINGPNNAHIQQVGDR.C
66	1216	1226	57.6	648.84	1295.66	1295.66	0	K.LLYNNVSNFGR.L
67	1227	1245	85.5	986.02	1970.03	1970.02	0	R.LASTLVHLGEYQAAVDGAR.K
68	1312	1326	86.4	856.43	1710.85	1710.85	0	R.AHMGMFTELAILYSK.F
69	1312	1326	47.5	864.43	1726.84	1726.84	0	R.AHMGMFTELAILYSK.F + Oxidation (M:3)
70	1312	1326	36.3	872.43	1742.84	1742.84	0	R.AHMGMFTELAILYSK.F + Oxidation (M:3.5)
71	1312	1326	45.3	864.43	1726.84	1726.84	0	R.AHMGMFTELAILYSK.F + Oxidation (M:5)
72	1332	1342	33.5	501.92	1502.75	1502.75	1	K.MREHLELFWSR.V
73	1334	1342	30.8	608.81	1215.60	1215.60	0	R.EHLELFWSR.V
74	1388	1397	18.7	589.83	1177.64	1177.63	1	K.EGQFKDIITK.V
75	1398	1406	55.4	563.8	1125.58	1125.58	0	K.VANVELYYR.A
76	1462	1481	82.5	1178.08	2354.14	2354.14	0	K.SVNESLNNLFITEEDYQALR.T
77	1499	1508	29.8	657.37	1312.71	1312.71	1	R.LEKHELIEFR.R
78	1502	1508	32.7	472.25	942.49	942.49	0	K.HELIEFR.R
79	1536	1545	48.1	565.25	1128.48	1128.48	0	K.DAMQYASESK.D
80	1589	1604	56.9	992.98	1983.94	1983.94	0	R.HNIMDFAMPYFIQVMK.E
81	1589	1604	57.1	1008.97	2015.93	2015.93	0	R.HNIMDFAMPYFIQVMK.E + Oxidation (M:4.8)
82	1610	1620	40	411.55	1231.64	1231.64	1	K.VDKLDASESLR.K
83	1613	1620	25.5	445.73	889.45	889.45	0	K.LDASESLR.K

CND3: 7 peptides. Peptide coverage: 8.7 %

Peptide	Start	End	Score	M/z (Obs)	Mr (Exp)	Mr (Calc)	Miss	Sequence
1	247	257	39.8	643.85	1285.68	1285.68	0	R.VMLLQQGLNDR.S
2	276	287	26.7	476.59	1426.76	1426.76	0	R.FSEGNILELLHR.L
3	387	398	58	673.33	1344.64	1344.64	0	R.GDFSYIGNLMTK.E
4	459	470	52.8	701.4	1400.79	1400.79	0	R.TQIVTEIISEIR.A

5	471	486	44.1	818.94	1635.86	1635.86	0	R.APIVTVGVNNDPADVR.K
6	695	708	61.3	811.92	1621.82	1621.82	0	K.LLSDFLDSEVSELR.T
7	718	728	59.3	605.34	1208.66	1208.66	0	K.LMFSGLLVSSR.I

COG3: 4 peptides. Peptide coverage: 6.5 %

Peptide	Start	End	Score	M/z (Obs)	Mr (Exp)	Mr (Calc)	Miss	Sequence
1	197	209	51.5	786.40	1570.79	1570.79	0	K.LSYFNELETINTK.L
2	265	280	23.7	912.51	1823.01	1823.00	0	K.TYTVNTLQTLTSQLLK.R
3	707	718	56.9	757.38	1512.75	1512.75	0	K.LFVEQLEEFMTK.V
4	733	745	40.2	759.39	1516.77	1516.77	0	K.YTLSQQPWAQPAK.V

COPG2:10 peptides. Peptide coverage: 13.2 %

Peptide	Start	End	Score	M/z (Obs)	Mr (Exp)	Mr (Calc)	Miss	Sequence
1	33	42	32.7	600.82	1199.63	1199.63	0	R.IFNETPINPR.R
2	76	85	24.1	611.32	1220.62	1220.61	0	R.LFQSNDQTLR.R
3	131	142	42	674.35	1346.69	1346.69	0	R.ITDGTMLQAIER.Y
4	339	350	68.3	622.90	1243.78	1243.78	0	R.SIATLAITTLLK.T
5	480	488	38.9	514.79	1027.57	1027.57	0	R.VVLENEAVR.A
6	578	590	37.3	715.89	1429.77	1429.76	0	K.SIPLAMAPVFEQK.A
7	591	602	36.1	650.38	1298.74	1298.74	0	K.AEITLVATKPEK.L
8	630	646	89.1	992.98	1983.95	1983.94	0	K.SSEPVQLTEAETEYFVR.C
9	821	832	22.6	459.91	1376.72	1376.72	0	K.NSHSLYLAGIFR.G
10	833	840	19.8	446.75	891.48	891.48	0	R.GGYDLLVR.S

Dyn2: 7 peptides. Peptide coverage: 9.5 %

Peptide	Start	End	Score	M/z (Obs)	Mr (Exp)	Mr (Calc)	Miss	Sequence
1	5	15	18.5	621.84	1241.67	1241.67	0	R.GMEELIPLVNK.L
2	45	54	30.4	554.29	1106.57	1106.57	0	K.SSVLENFVGR.D
3	114	123	36.8	533.33	1064.63	1064.63	0	K.GISPVPINLR.V
4	143	157	45.2	849.44	1696.87	1696.87	0	K.VPVGDQPPDIEYQIK.D
5	207	217	40.6	618.28	1234.55	1234.55	0	K.LDLMDEGTDAR.D
6	230	237	34.1	439.25	876.48	876.48	0	R.GYIGVVNR.S
7	344	361	55.4	902.44	1802.86	1802.86	0	R.IEGSGDQVDTLELSGGAR.I

EXOC2: 10 peptides. Peptide coverage: 12.1 %

Peptide	Start	End	Score	M/z (Obs)	Mr (Exp)	Mr (Calc)	Miss	Sequence
1	185	192	32.4	466.77	931.53	931.53	1	K.MAVTNLKR.Q
2	207	225	20.2	674.01	2019.01	2019.01	0	K.GGLSTFFEAQDALSAIHQK.L
3	226	241	35.5	861.92	1721.82	1721.81	1	K.LEADGTEKVEGSMTQK.L
4	242	248	31.5	429.25	856.48	856.48	0	K.LENVLNR.A
5	249	263	94.2	811.41	1620.81	1620.81	0	R.ASNTADTLFQEVLGR.K
6	272	279	28.3	464.27	926.53	926.53	0	R.NALNVLQR.F
7	282	292	22.9	688.39	1374.77	1374.77	0	K.FLFNLPLNIER.N
8	324	331	25.6	515.75	1029.48	1029.48	0	K.YYAEVETR.I
9	505	515	26.2	657.85	1313.68	1313.68	0	K.MIQEVMHSLVK.L
10	759	767	41.1	584.82	1167.62	1167.62	0	R.LFENYIELK.A

IP08: 8 peptides. Peptide coverage: 9.7 %

Peptide	Start	End	Score	M/z (Obs)	Mr (Exp)	Mr (Calc)	Miss	Sequence
1	20	31	64.6	690.34	1378.67	1378.67	0	R.IAAENELNQSYK.I
2	32	41	31.9	572.35	1142.68	1142.68	0	K.IINFAPSLLR.I
3	94	102	34.4	514.79	1027.57	1027.57	0	R.DNIVEGIIR.S
4	296	307	43.6	666.90	1331.78	1331.78	0	K.TYAVGIQQVLLK.I
5	553	565	41.7	752.86	1503.71	1503.71	0	R.ETENDDVTNVIQK.M
6	972	988	78	1003.99	2005.96	2005.96	0	R.DAAWYQLLMAPLSEDQR.T
7	989	1001	45.1	510.94	1529.78	1529.78	0	R.TALQEVYTLAEHR.R
8	1023	1037	31.7	762.38	1522.74	1522.74	0	K.GVLSAFNFGTVPSNN.-

TTI1: 3 peptides. Peptide coverage: 4.3 %

Peptide	Start	End	Score	M/z (Obs)	Mr (Exp)	Mr (Calc)	Miss	Sequence
1	24	36	61.1	777.90	1553.78	1553.78	0	K.TQTVENVEHLQTR.L
2	369	387	26.4	665.37	1993.08	1993.08	0	K.ALADILSESLHSLATSLPR.L
3	956	969	54.3	692.40	1382.79	1382.79	0	K.LAGSLVTQAPISAR.A

mTOR: 29 peptides. Peptide coverage: 13.8 %

Peptide	Start	End	Score	M/z (Obs)	Mr (Exp)	Mr (Calc)	Miss	Sequence
1	84	103	38.7	633.03	1896.08	1896.08	1	R.KGGILAIASLIGVEGGNATR.I
2	152	160	42.3	515.77	1029.52	1029.52	0	R.ALEWLGADR.N
3	261	273	28.4	487.64	1459.89	1459.89	0	R.IHGALLILNELVR.I
4	492	502	19.2	579.30	1156.59	1156.59	0	R.AMGPGIQQDIK.E
5	659	672	64.6	761.93	1521.84	1521.84	0	K.LLVVGITDPDPDIR.Y
6	733	748	22.5	609.33	1824.98	1824.98	0	K.MLIQILTELEHSGIGR.I + Oxidation (M:1)
7	756	766	54.9	605.82	1209.63	1209.63	0	R.MLGHLVSNAPR.L + Oxidation (M:1)
8	756	766	42.3	597.82	1193.63	1193.63	0	R.MLGHLVSNAPR.L
9	887	898	52.1	629.87	1257.73	1257.73	0	R.VLGLLGALDPYK.H
10	911	921	30.3	547.27	1092.53	1092.53	0	R.DASAVSLSESK.S
11	1081	1090	28.7	580.27	1158.52	1158.52	0	R.VFMHDNSPGR.I
12	1121	1132	63.5	656.85	1311.68	1311.68	0	K.LFDAPEAPLPSR.K
13	1142	1154	69.1	759.36	1516.70	1516.70	0	R.LTESLDFTDYASR.I
14	1162	1170	30.8	529.78	1057.54	1057.54	0	R.TLDQSPELR.S
15	1171	1186	37.2	849.44	1696.87	1696.87	0	R.STAMDTLSSLVFQLGK.K
16	1171	1186	49.2	857.44	1712.87	1712.87	0	R.STAMDTLSSLVFQLGK.K + Oxidation (M:4)
17	1188	1197	37.2	626.84	1251.67	1251.67	0	K.YQIFIPMVNK.V
18	1219	1235	25.1	683.66	2047.95	2047.95	0	K.GYTLADEEEDPLIYQHR.M
19	1239	1256	75	851.41	1700.81	1700.80	0	R.SGQGDALASGPVETGPMK.K
20	1278	1285	30.5	566.77	1131.53	1131.53	0	K.DDWLEWLR.R
21	1407	1422	47.9	833.97	1665.93	1665.93	0	K.GPTPAILESLISINNK.L
22	1501	1511	43	652.83	1303.64	1303.64	0	K.WTLVNDETQAK.M
23	1641	1651	24.2	635.32	1268.62	1268.62	0	R.SLVVSPHEDMR.T
24	1772	1784	62.7	776.87	1551.73	1551.73	0	K.VLQYYSAATEHDR.S
25	2135	2152	31.7	1006.03	2010.04	2010.04	0	R.DLELAVPGTYDPNQPIIR.I
26	2198	2217	56.6	734.74	2201.19	2201.19	0	R.VMQLFGLVNTLLANDPTSLR.K
27	2198	2217	30.4	740.07	2217.18	2217.18	0	R.VMQLFGLVNTLLANDPTSLR.K + Oxidation (M:2)
28	2284	2301	71.2	964.97	1927.93	1927.93	0	K.VEVFEHAVNNTAGDDLAK.L
29	2382	2397	57.8	892.92	1783.83	1783.82	0	R.MLTNAMEVTGLDGNYR.I

NCDN: 5 peptides. Peptide coverage: 8.2 %

Peptide	Start	End	Score	M/z (Obs)	Mr (Exp)	Mr (Calc)	Miss	Sequence
1	48	61	66.9	774.92	1547.82	1547.82	0	K.NDSEQFAALLLVTK.A
2	76	87	46.2	692.36	1382.70	1382.70	0	R.IFDAVGFTFPNR.L
3	130	140	36.5	616.37	1230.73	1230.73	0	K.IPILSTFLTAR.G
4	260	267	31.1	422.24	842.46	842.46	0	R.DLQAGLAR.I
5	579	593	62.2	749.92	1497.82	1497.82	0	R.LLSTSPALQGTPASR.G

NF1: 20 peptides. Peptide coverage: 8.8 %

Peptide	Start	End	Score	M/z (Obs)	Mr (Exp)	Mr (Calc)	Miss	Sequence
1	17	24	38.3	495.27	988.52	988.52	0	R.FDEQLPIK.T
2	207	218	67.3	642.88	1283.75	1283.74	0	K.VAQLAVINSLEK.A
3	249	261	77.5	721.36	1440.71	1440.71	0	K.LFDLVDGFAESTK.R
4	298	304	26	432.25	862.49	862.49	0	K.LFLDSLR.K
5	799	810	45.9	658.30	1314.59	1314.59	0	K.MEDGQAAESLHK.T
6	919	936	79.2	1010.54	2019.07	2019.08	0	K.DLVGLELSPALYPMLFNK.L
7	1177	1185	46.4	520.28	1038.54	1038.54	0	R.ATFMEVLTK.I
8	1326	1337	37.7	715.84	1429.67	1429.67	0	R.LEPSESLEENQR.N
9	1397	1412	62.3	847.94	1693.86	1693.86	0	R.FPQNSIGAVGSAMFLR.F
10	1413	1429	46.7	924.00	1845.99	1845.99	0	R.FINPAIVSPYEAGILDK.K
11	1589	1600	33.2	658.35	1314.68	1314.68	0	K.TLSIFYQAGTSK.A
12	1601	1611	45.1	635.83	1269.65	1269.65	0	K.AGNPIFYYVAR.R
13	1849	1870	83.7	1133.13	2264.24	2264.24	0	K.DVPGTLLNIALLNLGSSDPSLR.S
14	2151	2160	61.6	567.82	1133.63	1133.63	0	R.LSLTEFSLPK.F
15	2225	2235	32.1	723.36	1444.71	1444.71	0	K.WLDQWTELAQR.F
16	2355	2364	67.2	589.80	1177.58	1177.58	0	K.SPEEVFMAIR.N
17	2400	2408	19	474.27	946.53	946.53	0	R.HPSPAIVAR.T
18	2615	2628	74.9	748.50	1494.98	1494.97	0	K.IQALLLTVLATLVK.Y
19	2638	2652	63.4	871.47	1740.93	1740.93	0	R.ILYEYLAEASVVFPK.V
20	2721	2733	36.2	759.41	1516.81	1516.81	0	K.QTQIPDYAELIVK.F

NSF: 13 peptides. Peptide coverage: 19.5 %

Peptide	Start	End	Score	M/z (Obs)	Mr (Exp)	Mr (Calc)	Miss	Sequence
1	28	38	42.8	643.34	1284.66	1284.66	0	K.DFQSGQHVIVR.T
2	151	161	22.6	624.31	1246.61	1246.61	0	K.DIEAMDPSILK.G + Oxidation (M:5)
3	188	198	41.7	573.31	1144.61	1144.61	0	K.AENSSLNLIGK.A
4	284	293	22.8	541.81	1081.61	1081.61	0	K.VVNGPEILNK.Y
5	294	303	39.2	569.28	1136.55	1136.55	0	K.YVGESEANIR.K
6	305	314	56.1	604.28	1206.55	1206.55	0	K.LFADAEEEQR.R
7	393	401	27.2	516.26	1030.50	1030.50	0	K.MEIGLPDEK.G
8	416	427	48.5	648.35	1294.69	1294.69	0	R.GHQLLSADVDIK.E
9	517	529	67.2	729.40	1456.78	1456.78	0	R.VLDDGELLVQQTK.N
10	534	549	35.7	544.31	1629.91	1629.91	0	R.TPLVSVLLEGPPHSGK.T
11	595	607	40.9	760.38	1518.74	1518.73	0	K.SQLSCVVVDDIER.L
12	608	617	45	571.83	1141.65	1141.65	0	R.LLDYVPIGPR.F
13	640	648	32.2	487.31	972.60	972.60	0	K.LLIIGTTSR.K

PK3C3: 3 peptides. Peptide coverage: 4.3 %

Peptide	Start	End	Score	M/z (Obs)	Mr (Exp)	Mr (Calc)	Miss	Sequence
1	239	254	31.9	868.93	1735.85	1735.85	0	K.DGDESSPILTSFELVK.V
2	540	551	60.7	674.87	1347.72	1347.71	0	R.SLLAAQQTFVDR.L
3	573	582	25.5	550.80	1099.59	1099.59	0	R.LQALLGDNEK.M

RNBP6: 6 peptides. Peptide coverage: 6.7 %

Peptide	Start	End	Score	M/z (Obs)	Mr (Exp)	Mr (Calc)	Miss	Sequence
1	50	58	19.7	518.29	1034.57	1034.58	0	K.TTFLLDAVR.N
2	515	534	56.8	1072.59	2143.16	2143.16	0	K.LALEQLVTTIASVADTIEEK.F
3	535	547	19.4	810.41	1618.81	1618.81	0	K.FVPYYDIFMPSLK.H
4	535	547	59.1	818.41	1634.81	1634.81	0	K.FVPYYDIFMPSLK.H + Oxidation (M:9)
5	702	713	38.4	714.36	1426.70	1426.70	0	R.EGFVEYTEQVVK.L
6	764	783	54.3	1069.52	2137.03	2137.02	0	K.AIGTEPDTDVLSEIMNSFAK.S

RAPTOR: 5 peptides. Peptide coverage: 4.7 %

Peptide	Start	End	Score	M/z (Obs)	Mr (Exp)	Mr (Calc)	Miss	Sequence
1	339	348	27.4	581.34	1160.66	1160.66	0	R.QDLLVASLFR.N
2	604	616	54.6	751.42	1500.82	1500.82	0	K.LYSLLSDPIPEVR.C
3	850	867	90.9	908.97	1815.92	1815.92	0	R.VLDTSSLTQSAPASPTNK.G
4	1013	1021	41.9	567.30	1132.59	1132.59	0	R.LDDQIFLNR.N
5	1179	1191	39.6	629.36	1256.71	1256.71	0	R.SLIVAGLGDGSIR.V

SMG1: 6 peptides. Peptide coverage: 2 %

Peptide	Start	End	Score	M/z (Obs)	Mr (Exp)	Mr (Calc)	Miss	Sequence
1	1629	1640	21.2	615.81	1229.60	1229.60	0	K.VVDNASQGEGVR.L
2	2022	2034	24.3	676.35	1350.68	1350.68	0	R.SITAAPAETPHEK.W
3	2532	2543	31.6	759.87	1517.72	1517.72	0	R.YSEHTQLQTQQR.A
4	3011	3020	32.7	633.36	1264.71	1264.71	0	R.QVLEEIFFLK.R
5	3246	3256	48.5	608.83	1215.65	1215.65	0	K.LAALESSIEQR.L
6	3312	3327	83.9	866.48	1730.94	1730.95	0	R.TAEALNLDAALFELIK.R

TBCD: 10 peptides. Peptide coverage: 11.2 %

Peptide	Start	End	Score	M/z (Obs)	Mr (Exp)	Mr (Calc)	Miss	Sequence
1	170	182	32.6	691.87	1381.73	1381.73	0	R.LDGNLLTQPGQAR.M
2	189	202	66.3	796.45	1590.89	1590.89	0	R.ILQIAESYLIVSDK.A
3	205	213	33.6	451.26	900.5	900.5	0	R.DAAAVLVSR.F
4	361	370	19	556.36	1110.7	1110.7	0	R.VIEQLLVGLK.D
5	509	520	44.9	610.8	1219.59	1219.59	0	R.AASAAFQENVGR.Q
6	635	656	21.5	811.42	2431.25	2431.25	0	K.LAAQENRPVTDHLDEQAVQGLK.Q
7	791	799	50.9	514.32	1026.62	1026.62	0	R.LQQVLTGLR.A
8	800	815	74.3	866.91	1731.81	1731.81	0	R.AVTHTSPEDVSFAESR.R
9	952	968	79.7	909.94	1817.87	1817.87	0	R.SDVASVNWSAPSQAFPR.I
10	1000	1010	45.2	685.82	1369.63	1369.63	0	R.HSTQSLFEYMK.G

TELO2: 9 peptides. Peptide coverage: 11.8 %

Peptide	Start	End	Score	M/z (Obs)	Mr (Exp)	Mr (Calc)	Miss	Sequence
1	133	142	36.4	595.8	1189.59	1189.59	0	R.LAVLMEAQCR.Q
2	143	153	18.4	650.87	1299.73	1299.73	0	R.QQTQPGFILLR.E

3	187	194	27.5	457.77	913.52	913.52	0	R.LLGEEVVR.V
4	255	262	21.6	478.26	954.51	954.51	0	R.LVEQVPDR.A
5	303	309	16.1	438.77	875.52	875.52	0	K.LLFLQSR.L
6	338	350	65	724.87	1447.73	1447.73	0	K.ELLETWGSSSAIR.H
7	405	416	20.6	630.85	1259.69	1259.69	0	R.LGMIVAEVVSAR.I + Oxidation (M:3)
8	517	530	67.2	819.37	1636.73	1636.72	0	R.DCVEALTTSEDIER.W
9	799	814	19.5	635.95	1904.82	1904.82	1	R.SWLADVAEKDPDEDCR.T

TNPO1: 10 peptides. Peptide coverage: 14.7 %

Peptide	Start	End	Score	M/z (Obs)	Mr (Exp)	Mr (Calc)	Miss	Sequence
1	45	64	55.7	1236.64	2471.27	2471.27	0	K.LEQLNQYPDFNNYLIFVLTK.L
2	116	128	84.1	644.40	1286.78	1286.78	0	R.ATVGILITTIASK.G
3	129	140	23.4	705.38	1408.73	1408.73	0	K.GELQNWPDLLPK.L
4	246	254	56.9	530.32	1058.62	1058.62	0	R.ALVMLLEVR.M + Oxidation (M:4)
5	258	272	19	632.00	1892.98	1892.98	0	R.LLPHMHNIVEYMLQR.T
6	318	327	39.3	596.85	1191.67	1191.68	0	K.YSDIDIILLK.G
7	328	346	32.6	1100.50	2198.99	2198.99	0	K.GDVEEDETIPDSEQDIRPR.F
8	693	704	39.7	640.35	1278.68	1278.68	0	R.QSSFALLGDLTK.A
9	774	785	50.1	651.38	1300.73	1300.73	0	K.TLLENTAITIGR.L
10	880	889	22.9	596.33	1190.64	1190.63	0	R.FSDQFPLPLK.E

XPO4: 17 peptides. Peptide coverage: 15.6 %

Peptide	Start	End	Score	M/z (Obs)	Mr (Exp)	Mr (Calc)	Miss	Sequence
1	21	34	23	793.39	1584.78	1584.78	0	K.VLMAPPSMVNNEQR.Q
2	21	34	36.5	809.39	1616.76	1616.77	0	K.VLMAPPSMVNNEQR.Q + Oxidation (M:3.8)
3	21	34	37.4	801.39	1600.77	1600.77	0	K.VLMAPPSMVNNEQR.Q + Oxidation (M:8)
4	189	196	22.4	518.26	1034.50	1034.50	0	R.VFQEEDLR.Q
5	459	486	26.8	1075.82	3224.45	3224.44	1	R.EEEEISELQEDDRDQFSDQLASVGMLGR.I
6	514	530	63	905.46	1808.90	1808.90	0	R.HQQQLLASPGSSTVDNK.M
7	624	636	18.6	713.87	1425.73	1425.73	0	R.ADLTHLLSPQMGK.D + Oxidation (M:11)
8	648	655	22	490.77	979.52	979.52	0	K.TYLLVDEK.L
9	737	749	72.6	721.39	1440.77	1440.77	0	R.SPPLNFLSSPVQR.T
10	754	768	51.2	795.40	1588.79	1588.79	0	K.ALVLGGFAHMDTETK.Q
11	754	768	34.9	803.40	1604.79	1604.79	0	K.ALVLGGFAHMDTETK.Q + Oxidation (M:10)
12	769	782	50	908.97	1815.93	1815.93	0	K.QQYWTEVLQPLQQR.F
13	925	937	88.2	810.37	1618.72	1618.72	0	K.EFIDFSDTDEVFR.G
14	1039	1051	54.5	724.87	1447.73	1447.73	0	K.AQETDSPLFLATR.H
15	1056	1065	29.5	603.35	1204.69	1204.69	0	K.LVFDMLVLQK.H
16	1116	1128	37.4	693.88	1385.75	1385.75	1	K.LTASSTPPTLDRK.Q
17	1116	1127	31.6	629.84	1257.66	1257.66	0	K.LTASSTPPTLDR.K

XPOT: 25 peptides. Peptide coverage: 25.6 %

Peptide	Start	End	Score	M/z (Obs)	Mr (Exp)	Mr (Calc)	Miss	Sequence
1	21	29	45.7	541.80	1081.58	1081.58	0	R.ALAYFEQLK.I
2	30	45	65.1	907.95	1813.88	1813.88	0	K.ISPDAWQVCAEALAQR.T
3	54	65	16.7	527.93	1580.78	1580.78	0	K.FFCFQVLEHQVK.Y
4	68	80	53.9	789.93	1577.84	1577.84	0	K.YSELTTVQQQLIR.E
5	138	144	35.4	418.24	834.46	834.46	0	R.GVDLYLR.I
6	145	156	55.6	695.87	1389.72	1389.72	0	R.ILMAIDSELVDR.D + Oxidation (M:3)
7	145	156	45.9	687.87	1373.72	1373.72	0	R.ILMAIDSELVDR.D
8	241	253	35.7	806.85	1611.69	1611.69	0	R.EEACDCLFEVVNK.G
9	317	328	69.1	658.35	1314.68	1314.68	0	K.NAQEALQAIETK.V
10	360	369	20.6	580.32	1158.62	1158.62	0	K.QLTVLSDQQK.A
11	370	381	46.6	661.35	1320.68	1320.68	0	K.ANVEAIMLAVMK.K + Oxidation (M:7.11)
12	370	381	70.9	645.35	1288.69	1288.69	0	K.ANVEAIMLAVMK.K
13	415	428	30.9	748.45	1494.88	1494.88	0	R.LAQVSPELLLASVR.R
14	429	442	17.1	575.30	1722.88	1722.88	1	R.RVFSSTLQNWQTTR.F
15	430	442	54.1	784.40	1566.78	1566.78	0	R.VFSSTLQNWQTTR.F
16	443	451	47.7	555.29	1108.56	1108.56	0	R.FMEVEVAIR.L + Oxidation (M:2)
17	475	483	42.2	511.74	1021.47	1021.47	0	K.ASALQDMMR.T
18	558	569	20.2	753.38	1504.74	1504.73	0	K.QMNPFIEDILNR.I + Oxidation (M:2)
19	558	569	23.8	745.38	1488.74	1488.74	0	K.QMNPFIEDILNR.I
20	619	627	26.1	529.80	1057.58	1057.58	0	R.NLLTPLMEK.F
21	635	643	27.4	560.77	1119.52	1119.52	0	K.LMLAQDEER.Q + Oxidation (M:2)
22	738	752	30.3	871.99	1741.96	1741.96	0	K.DLQEFIPLINQITAK.F
23	825	843	49.6	1020.57	2039.13	2039.13	0	R.VLVTVIQGAVEYPDPIAQK.T
24	844	851	36.8	491.28	980.54	980.54	0	K.TCFIILSK.L
25	860	871	43.1	657.82	1313.63	1313.63	0	K.DGPVGFADFVYK.H

For each peptide identified, the position in protein sequence, mascot score, experimental and calculated masses, number of trypsin missed cleavage and sequence are indicated.
